# Modelling C-arm fluoroscopy and operating table kinematics via machine learning

**DOI:** 10.3389/frobt.2025.1691576

**Published:** 2026-02-05

**Authors:** Faria Jaheen, Vinod Gutta, Pascal Fallavollita

**Affiliations:** 1 School of Electrical Engineering and Computer Science, University of Ottawa, Ottawa, ON, Canada; 2 Interdisciplinary School of Health Sciences, University of Ottawa, Ottawa, ON, Canada

**Keywords:** C-arm fluoroscopy, operating table, machine learning, forward kinematics, inverse kinematics, surgery

## Abstract

This work presents a machine learning driven framework for data-efficient kinematic modeling and workspace optimization in modular C-arm fluoroscopy systems integrated with operating tables. A comprehensive dataset of joint configurations and end-effector poses annotated with voxelized collision status enables the training of predictive models across multiple system configurations ranging from 5 to 9 degrees of freedom. Leveraging expansive simulation-derived datasets, as well as clinical assessment through simulated X-ray generation, the models are trained and validated, achieving sub-millimetric positional accuracy and sub-degree angular precision while delivering real-time inference that surpasses conventional methods in scalability, robustness, and computational latency. The proposed framework demonstrates the viability of data-driven trajectory planning in multi-degree of freedom C-arm systems, providing a clinically relevant solution for improving imaging access and reducing intraoperative collision risks.

## 1 Introduction

C-arm fluoroscopy systems are central to intraoperative imaging, particularly in minimally invasive surgeries. Precise and collision-free positioning of the C-arm is critical for ensuring optimal anatomical visualization while minimizing radiation exposure and procedural time. However, conventional C-arm systems are typically limited in their degrees of freedom (DoF), constraining the reachable workspace and reducing imaging flexibility during complex procedures. The clinical demand for adaptive and precise maneuvering has led to the integration of operating tables with C-arm systems, creating kinematically coupled, redundant configurations that must be coordinated in real time. When considered together, the C-arm and operating table form an integrated medical robotic system whose safe and efficient motion planning directly impacts patient safety and clinical workflow efficiency.

Traditional analytical kinematic approaches, while mathematically elegant, encounter serious challenges in these high DoF, nonlinear environments. Analytical solvers for forward and inverse kinematics (FK and IK) depend on geometric or algebraic formulations that are tractable only for low-DoF serial manipulators (Siciliano et al., 2008; Craig, 2005). As the number of DoFs increases such as in a modular C-arm with the operating table the system exhibits nonlinear coupling, parameter redundancy, and joint interdependence that make closed-form IK derivations intractable. Furthermore, the presence of joint limits, collision constraints, and complex motion paths further limits the scalability and robustness of symbolic solutions in real-time clinical applications. These limitations underscore the need for learning-based, data-driven modeling frameworks capable of generalizing across diverse surgical poses and hardware configurations.

Recent developments in machine learning (ML) have demonstrated the capacity of data-driven models to approximate complex kinematic mappings with high fidelity, enabling more flexible and adaptive motion control. Supervised learning methods such as deep neural networks (DNNs) and ensemble models can efficiently learn nonlinear mappings between Cartesian and joint spaces, capturing intricate interdependencies that analytical methods often neglect (Xiao et al., 2023; Hsieh and Hou, 2024). For instance, Xiao et al. (2023) employed a Long Short-Term Memory (LSTM) network to model the IK of tendon-driven surgical manipulators, mitigating nonlinear effects such as cable slack and hysteresis. Relaño et al. (2023) leveraged Gaussian Process (GP) regression and its Deep GP variant to model soft robotic arms, providing uncertainty quantification and improved generalization compared to traditional solvers. Similarly, Kinoshita et al. (2008) developed a Newton-like Inverse Kinematics (IK) approach based on ridge regression with adaptive regularization to achieve faster convergence while maintaining accuracy. In rehabilitation robotics, Shah et al. (2024) used a DNN-based framework for upper-limb exoskeletons, achieving superior joint angle prediction compared to analytical solvers. Extending this trend, Hsieh and Hou, (2024) proposed a model-free, end-to-end control approach for multi-input multi-output (MIMO) robotic systems, eliminating the need for symbolic modeling and emphasizing data-driven motion learning.

Parallel advances have emerged in forward kinematics (FK) modeling, particularly in redundant or continuum systems where analytical FK is similarly cumbersome. Cho et al. (2024) developed a deep decoder neural network to reconstruct tendon-driven continuum robot shapes from point clouds, achieving improved accuracy over physics-based models by capturing hysteresis. Singh et al. (2024) compared analytical, geometric, numerical, and AI-based FK solvers in a 6-axis humanoid arm, demonstrating the superior accuracy and adaptability of deep learning under nonlinear motion conditions. Beyond regression modeling, dimensionality reduction techniques have enhanced both learning efficiency and model interpretability. Shimizu et al. (2018) used Functional Principal Component Analysis (FPCA) to synthesize humanoid motions in low-dimensional manifolds while preserving dynamic feasibility. In visual localization, Carreira et al. (2012) and Tamimi and Zell, (2004) applied PCA and Kernel PCA to achieve real-time, noise-robust localization in unstructured environments. Similarly, Ra et al. (2008) integrated PCA-guided genetic optimization to evolve energy-efficient humanoid trajectories within feasible kinematic constraints.

Beyond these deep and statistical learning methods, several studies have explored optimization-enhanced neural architectures that integrate metaheuristic algorithms for kinematic modeling. Such approaches aim to improve ANN convergence and hyperparameter tuning efficiency in robotic manipulators of lower dimensionality. For instance, metaheuristic-driven inverse kinematic frameworks have been proposed for 2-DoF and 3-DoF exoskeletons using hybrid ANN-optimization models (Bouzid et al., 2025a) and (Bouzid et al., 2025b) respectively, while SCARA manipulator studies have examined both forward and inverse kinematics through ANNs trained with diverse optimizers and dataset variations (Bouzid et al., 2024a) and (Bouzid et al., 2024b) correspondingly. These works underscore the effectiveness of coupling neural learning with evolutionary or swarm-based optimization to enhance local accuracy and training robustness. However, their applicability remains confined to low-DoF laboratory platforms, without addressing the scalability and coupling complexities inherent in high-DoF clinical robotic systems such as modular C-arms integrated with multi-DoF operating tables.

Collectively, these studies mark a paradigm shift from symbolic to intelligent, data-driven kinematic modeling. Yet, despite this progress, existing works focus on industrial manipulators, continuum robots, or rehabilitation devices, with limited attention to modular C-arm systems used in surgical imaging. None of the prior approaches have developed a unified framework that simultaneously models forward and inverse kinematics for a modular C-arm fluoroscopy system coupled with an operating table, while analyzing performance scalability across multiple DoF configurations.

To bridge this gap, the present study introduces a unified machine-learning framework for forward and inverse kinematic modeling of a modular C-arm fluoroscopy system integrated with a mobile operating table. The system comprises a 6-DoF enhanced OEC Elite C-arm augmented by lateral translation coupled with a 3-DoF STERIS CMAX surgical table, forming a complete 9-DoF robotic imaging platform. A large-scale dataset containing over 131,000 collision-free joint configurations and corresponding 6D Cartesian poses was generated in MATLAB/Simulink and Blender. Five supervised learning models are trained and evaluated to establish accurate bidirectional mappings between joint and task spaces. The proposed framework demonstrates high modeling accuracy within clinical tolerance, robustness under noisy and perturbed data, and scalability across 5–9 DoF configurations, thereby offering a foundation for intelligent, real-time motion planning in intraoperative imaging systems.

## 2 Integrated C-arm fluoroscope and operating table degrees of freedom

### 2.1 Description of OEC 3D C-arm and CMAX™ X-ray operating table

**FIGURE 1:**
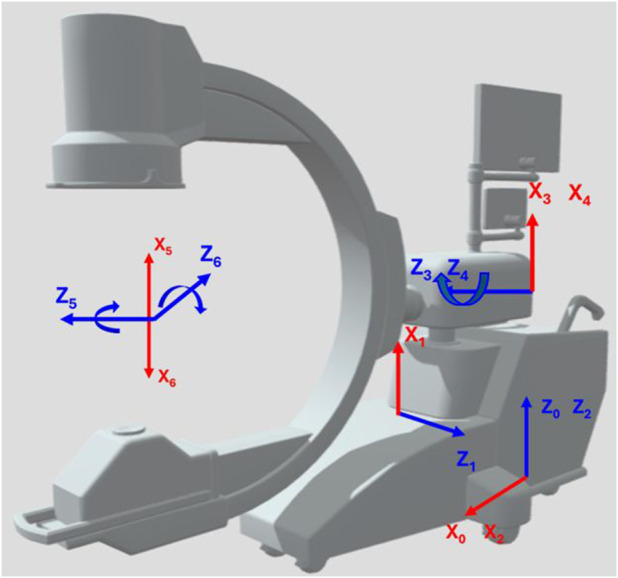
Joints of a C-arm fluoroscope. We modeled the C-arm using the manufacturing specifications of the GE Healthcare OEC 3D C-arm. Adapted from C Arm Xray Device by laurentiubodisteanu, licensed under CC BY 4.0, source: https://sketchfab.com/3d-models/c-arm-xray-device-08e65d9a5caa4dcf9338e1b980a9ec00

The imaging system analyzed in this paper consists of the GE OEC 3D C-arm integrated with the Steris CMAX™ X-ray Operating table, combining translational and rotational mobility to enhance intraoperative imaging flexibility. We note here that any other C-arm device and operating room model can be used for the proposed methodology as well as their respective range of motion specifications determined by the manufacturers. The GE OEC 3D C-arm system is a mobile fluoroscopic imaging platform characterized by six degrees of freedom comprising both translational and rotational movements as shown in Figure 1.

The C-arm architecture integrates three prismatic joints and three revolute joints, enabling precise manipulation of the imaging source and detector around the patient. The prismatic joints include the C-arm Lateral translation, with a symmetric range of ±0.5 m; the C-arm Vertical translation, permitting vertical displacement between 0 and 0.46 m; and the C-arm Horizontal translation, allowing forward motion up to 0.15 m. Rotational mobility is achieved through the revolute joints: the C-arm Wigwag rotation provides yaw adjustments within ±10°, the C-arm Tilt rotation supports extensive pitch articulation from −90° to 270°, and the C-arm Orbital rotation enables roll motion from −100° to 100°.

**FIGURE 2:**
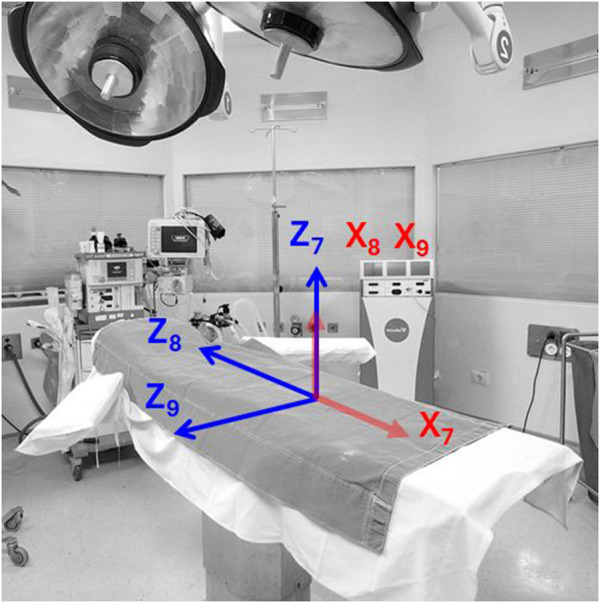
Joints of an operating table. We modeled the operating table using the manufacturing specifications of the Steris CMAX™ X-ray Operating table. Adapted from Hospital Operating Room by Vithas, in the Public Domain, source: https://www.needpix.com/photo/1133954/

The CMAX™ X-ray Operating table (STERIS Corporation, 2023) augments the operational flexibility of the C-arm imaging system by providing three additional prismatic degrees of freedom for patient positioning, as illustrated in Figure 2. The Table Vertical joint provides vertical elevation from 0 to 0.36 m, the Table Longitudinal joint allows translation along the head-to-foot axis with a range of 0–0.7 m, and the Table Transverse joint supports lateral motion from −0.13 to 0.13 m. Although the CMAX™ table is mechanically equipped with rotational capabilities (table roll and yaw), these two rotational degrees of freedom are deliberately excluded from the system model to preserve patient stability and ensure procedural safety. Rotational adjustments during surgery may increase the risk of inadvertent patient movement, and potentially disrupt the precise alignment required for image-guided interventions or delicate anatomical access.

The joint limits of the GE OEC 3D C-arm and CMAX™ X-ray Operating table define the operational workspace and mechanical boundaries of the integrated system. These constraints were rigorously embedded within the kinematic modeling, workspace analysis, and inverse kinematics computations to ensure that all predicted and simulated configurations remained within the physically realizable domain. By strictly enforcing these limits, the analysis guarantees mechanical feasibility across all modular C-arm configurations, supporting reliable and clinically deployable motion planning strategies. The integration of the C-arm’s combined translational and rotational degrees of freedom with the operating table’s prismatic motions substantially enlarges the effective surgical workspace enabling adaptive positioning across diverse anatomical targets. This expanded mobility supports efficient intraoperative imaging across complex trajectories, optimizing access and visualization while upholding procedural safety and spatial precision. The Denavit–Hartenberg (DH) parameters defining the kinematic structure of the combined GE OEC 3D C-arm and CMAX™ X-ray Operating table are presented in Table 1. These parameters serve as the foundational representation for the system’s forward and inverse kinematics formulations, capturing the joints of the C-arm and the operating table within a unified kinematic model.

**TABLE 1:** DH Parameterization of the GE OEC 3D C-arm and CMAX™ X-ray operating table.

Joint	Link length, $a_{i}$ (m)	Link twist, $\alpha_{i}$ (rad)	Link offset, $d_{i}$ (m)	Joint angle, $\theta_{i}$ (rad)	Range
C-arm lateral	0	0	$d_{1}$	0	−0.5 m ∼ 0.5 m
C-arm vertical	0	−π2	$d_{2}$	0	0 ∼ 0.46 m
C-arm wigwag	0	π2	0	$\theta_{3}$	−10° ∼ 10°
C-arm horizontal	0	0	$d_{4}$	0	0 ∼ 0.15 m
C-arm tilt	0	−π2	0	$\theta_{5}$	−90° ∼ 270°
C-arm orbital	0	π2	0	$\theta_{6}$	−100° ∼ 100°
Table vertical	0	0	$d_{7}$	0	0 ∼ 0.36 m
Table longitudinal	0	−π2	$d_{8}$	0	0 ∼ 0.7 m
Table transverse	0	π2	$d_{9}$	0	−0.13 m ∼ 0.13 m

### 2.2 Joint annotation and system configuration

To standardize kinematic modeling, Table 2 summarizes the abbreviations, joint types, and functional descriptions of all joints used in the C-arm and integrated operating table system. Six degrees of freedom are attributed to the C-arm itself, comprising four prismatic joints (lateral, vertical, horizontal) and two revolute joints (wigwag, tilt, orbital). The integrated operating table contributes up to three additional prismatic joints: vertical lift, longitudinal slide, and transverse slide. This nomenclature facilitates consistent formulation of workspace analysis and inverse kinematics across configurations ranging from 5DoF to 9DoF.

**TABLE 2:** Summary of joint abbreviations, joint types, and functional descriptions for the C-arm and integrated operating table.

Joint name	Abbreviation	Joint type	Description
C-arm lateral	CL	Prismatic (P)	Lateral translation along X-axis
C-arm vertical	CV	Prismatic (P)	Vertical translation along Z-axis
C-arm wigwag	CW	Revolute (R)	Axial rotation around patient (yaw rotation)
C-arm horizontal	CH	Prismatic (P)	Depth translation along Y-axis
C-arm tilt	CT	Revolute (R)	Tilt up/down (pitch rotation)
C-arm orbital	CO	Revolute (R)	Orbital rotation around patient longitudinal axis (roll rotation)
Table vertical	TV	Prismatic (P)	Elevation of the operating table (vertical lift)
Table longitudinal	TL	Prismatic (P)	Translation along patient head-foot direction (longitudinal axis)
Table transverse	TT	Prismatic (P)	Lateral translation of table (sideways motion)

## 3 Methodology

**FIGURE 3:**
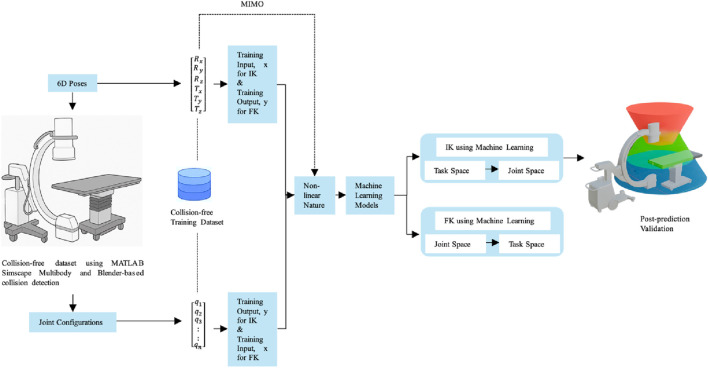
Kinematic learning framework.

The proposed kinematic learning framework as shown in Figure 3 employs a Multiple-Input, Multiple-Output (MIMO) modeling strategy to simultaneously predict high-dimensional robotic variables within the modular C-arm and surgical table system. This approach is structured into four key stages. In 1st stage, collision-free dataset generation is conducted using MATLAB Simscape Multibody and Blender-based collision detection pipelines. Datasets are created for five system configurations (ranging from 5 to 9 DoF), each comprising valid joint combinations and their corresponding 6D end-effector poses (translation and rotation). For each configuration, forward mapping pairs joint configurations as input with 6D poses as output, while inverse mapping pairs 6D poses as input with joint variables as output. In the second stage, the MIMO model formulation replaces traditional single output regressors with a unified multi-output regression framework. In the forward kinematics task, the model learns a mapping from the joint vector $q\in R^{n}$ to a 6D pose vector $x=\left[R_{x},R_{y},R_{z},T_{x},T_{y},T_{z}\right]\in R^{6}$ .

Conversely, for inverse kinematics, the model takes the 6D pose as input and predicts up to nine joint values simultaneously. This formulation captures spatial and kinematic dependencies across joints, allowing the model to learn interactions between coupled prismatic and revolute motions. In the third stage, model training and validation are performed using five supervised learning algorithms: Ridge Regression, K-Nearest Neighbors, Random Forest, Gradient Boosting Machines, and Deep Neural Networks (DNNs). All models are configured for multi-output regression, and training is performed on a standardized 70/15/15 train-validation-test split. Hyperparameter optimization is conducted via 5-fold cross-validation to ensure generalizability. For DNNs, Principal Component Analysis (PCA) is optionally applied to improve numerical conditioning and reduce input redundancy. Finally, a comprehensive evaluation and comparative analysis is conducted using four regression metrics: MAE, MSE, RMSE, and *R* ^2^. These metrics are computed both per output and across all outputs to assess fine-grained performance and overall system accuracy. Evaluation results are visualized through joint-specific heatmaps and pose-level prediction plots enabling model-level and DoF-level insight. This structured MIMO-based learning framework not only ensures consistency across predicted joint configurations or poses but also reduces training complexity compared to isolated regressors. Its joint-aware architecture facilitates robust and interpretable kinematic learning for modular robotic systems operating in safety-critical surgical environments.

### 3.1 Collection of collision-free pose datasets

A comprehensive dataset generation pipeline was developed to support machine learning–based inverse kinematics (IK) prediction for modular C-arm systems with integrated surgical table mobility. The pipeline combines analytical forward kinematics using MATLAB Simscape Multibody with geometric collision detection in Blender, ensuring that all joint configurations used in learning are both mechanically feasible and clinically safe. Kinematic models for five system configurations ranging from 5 to 9 degrees of freedom (DoF) were constructed in Simscape based on the Denavit–Hartenberg (DH) parameterization of the GE OEC 3D C-arm and the CMAX™ X-ray Surgical Table. These configurations included a combination of prismatic and revolute joints representing translational and rotational motions of the C-arm and translational mobility of the surgical table. Each joint was constrained within experimentally validated motion limits. For every configuration, forward kinematics were computed in MATLAB to produce corresponding end-effector poses in task space represented by translation vectors (t_x_, t_y_, t_z_) and Euler angles (r_x_, r_y_, r_z_).

To evaluate the physical feasibility of each configuration, the computed joint values and associated kinematic transforms were exported to Blender, where high-fidelity 3D mesh models of the C-arm and surgical table were used to perform collision detection. In this simulation environment, a pose was classified as “colliding” if any portion of the C-arm mesh (e.g., image intensifier, gantry frame, X-ray source) intersected with the table geometry. Collision detection was automated using Blender’s physics engine and scripted logic that flagged any geometric intersections. Only configurations confirmed to be entirely collision-free were retained for downstream use. The original simulation workflow generated three types of samples: collision-prone, mixed-overlap, and collision-free. However, this study deliberately selected only the collision-free subset to ensure that all machine learning models are trained on physically valid data, reducing the risk of unsafe pose prediction at deployment.

The final datasets encompass five system configurations spanning from a Conventional C-arm (5-DoF) to an Enhanced C-arm (6-DoF) with additional lateral translation, and integrated table systems offering 7-DoF, 8-DoF, and 9-DoF motion. The datasets comprise 131,229 poses for the 9-DoF system (C-arm combined with vertical, longitudinal, and transverse table translations), 96,257 for the 8-DoF system (excluding longitudinal movement), 41,173 for the 7-DoF system (only transverse table translation), 20,000 for the Enhanced 6-DoF C-arm, and 7,209 poses for the Conventional 5-DoF C-arm. The total sample sizes of collision-free pose datasets are summarized in Table 3.

**TABLE 3:** The total sample sizes of collision-free pose datasets generated for C-arm and integrated operating table configurations across varying degrees of freedom.

Configuration	Total degrees of freedom	Number of collision-free poses
C-arm + table vertical, longitudinal and transverse	9DoF	131229
C-arm + table vertical and transverse	8DoF	96257
C-arm + table transverse	7DoF	41173
Enhanced C-arm (with added lateral motion)	6DoF	20000
Conventional C-arm (standard model)	5DoF	7209

### 3.2 Dataset structure for machine learning-based inverse kinematics

The dataset used for machine learning-based inverse kinematics comprises sequentially indexed entries (SI. No.), with each entry containing the active joint configuration and its corresponding end-effector pose. These poses calculated using forward kinematics are expressed as 6D transformations consisting of Euler angles (R_x_, R_z_, R_z_) for orientation and Cartesian translations (T_x_, T_y_, T_z_) for position. This representation captures the full spatial configuration of the C-arm imaging system in relation to the surgical field. Table 4 presents the complete dataset structure for the 9DoF configuration, which includes six degrees of freedom from the C-arm (CL, CV, CW, CH, CT, CO) and three translational degrees of freedom from the integrated mobile operating table (TV, TL, TT). This dataset is used within the paper to illustrate the full joint-to-pose mapping and serves as the primary benchmark for model training and evaluation. For brevity and clarity, datasets corresponding to the 8DoF, 7DoF, enhanced 6DoF, and conventional 5DoF configurations are not included directly in the manuscript. However, these datasets follow the same structural format as Table 5, with appropriate exclusion of joints (e.g., TL, TT, CL) based on the respective DoF constraints. These additional datasets, along with the 9DoF dataset are publicly available in a dedicated GitHub repository (Jaheen, 2025).

**TABLE 4:** Dataset of a 9DoF C-arm and integrated operating table.

SI. No.	CL	CV	CW	CH	CT	CO	TV	TL	TT	R_x_	R_y_	R_z_	T_x_	T_y_	T_z_
1	9	1	7	1	180	0	35	70	−11	−83	0.00	−90	−3	−18.63	−25.60
2	11	2	6	0	180	0	36	54	−12	−84	0.00	−90	−3	−20.32	−14.32
3	−12	1	0	0	180	0	34	60	−13	−90	0.00	−90	−2	−20.50	−13.00
.	.	.	.	.	.	.	.	.	.	.	.	.	.	.	.
.	.	.	.	.	.	.	.	.	.	.	.	.	.	.	.
.	.	.	.	.	.	.	.	.	.	.	.	.	.	.	.
131227	−49	46	3	10	0	−35	16	6	0	87.54	1.72	124.96	61	2.28	86.37
131228	−41	30	10	9	0	−35	0	10	3	81.78	5.72	124.59	61	2.08	93.61
131229	−42	30	7	6	0	−35	0	5	5	84.26	4.01	124.80	61	2.34	91.01

**TABLE 5:** Optimized hyperparameters for each machine learning model across multiple degrees of freedom.

Model	Hyperparameter	DoF	Rx	Ry	Rz	Tx	Ty	Tz
Linear regression	Regularization strength	9	1	1	1	0.01	0.01	0.1
		8	1	1	1	0.01	0.01	0.01
		7	1	1	1	0.01	0.01	0.01
		6	0.1	0.1	0.01	0.01	0.01	0.01
		5	0.01	0.1	0.01	0.01	0.01	0.01
Gradient boosting machine	Learning rate	9	0.2	0.2	0.1	0.2	0.2	0.1
		8	0.2	0.2	0.2	0.2	0.2	0.2
		7	0.2	0.1	0.2	0.2	0.1	0.1
		6	0.2	0.2	0.2	0.2	0.1	0.1
		5	0.1	0.2	0.2	0.2	0.1	0.1
K-nearest neighbors	Number of nearest neighbors	9	9	5	9	9	9	9
		8	9	3	7	9	9	9
		7	7	3	7	9	9	9
		6	3	9	5	9	7	9
		5	3	7	9	5	9	5
Random forest	Number of decision trees	9	100	100	300	300	300	300
		8	100	300	200	300	300	300
		7	200	300	200	100	300	300
		6	200	100	100	100	300	300
		5	300	100	300	100	300	200
Deep neural network	Number of layers, activation function number of epochs, batch size	All	Layers = [256, 128, 64], Activation function = “ReLU”, Optimizer = “adam”, Epochs = 300, Batch size = 32					

### 3.3 Nonlinearity, redundancy, and singularity handling

The integrated C-arm and operating table system exhibits a highly nonlinear and redundant kinematic structure because translational (prismatic) and rotational (revolute) joints are coupled through sequential trigonometric transformations. This results in a non-linear mapping between joint variables, and the end-effector pose, where multiple joint configurations can realize identical Cartesian positions and orientations.

All singularity analyses and redundancy-resolution strategies were established in earlier works by Jaheen et al. (2025a), Jaheen et al. (2025b). During dataset generation for this study, singularity-prone and collision-inducing configurations were excluded, ensuring that the training data represent only mechanically feasible, continuous trajectories. The inverse-kinematics stage employed a BFGS gradient-projection algorithm to select the configuration closest to the preceding valid state, thereby maintaining continuity across the redundant workspace.

For rotational representation, the end-effector attitude was initially parameterized in unit-quaternion form and subsequently transformed into XYZ Euler angles following a consistent roll–pitch–yaw convention. A wrap-angle normalization within the range [–180°, 180°] was applied to preclude discontinuities at angular boundaries, thus maintaining orientation coherence throughout both data preprocessing and model evaluation. Consequently, the datasets utilized for learning realistically encapsulate the intrinsic nonlinear behavior of the C-arm and operating table mechanism while remaining singularity-free, dynamically coherent, and kinematically consistent across the entire workspace.

### 3.4 Machine-learning framework

To approximate the highly nonlinear and redundant mapping between joint-space variables and end-effector poses of the integrated C-arm–table system, a supervised ML framework was developed and trained on the singularity-free, collision-free datasets generated from the preceding inverse-kinematics pipeline. The objective was to construct predictive models capable of learning the deterministic, continuous relationship between the joint configuration vector and the corresponding Cartesian pose representation. Multiple regression architectures were investigated to capture this mapping at varying levels of model complexity, including Ridge Regression, K-Nearest Neighbors (KNN), Random Forest (RF), Gradient Boosting Regression (GBR), and Deep Neural Networks (DNN). Each model was independently optimized through cross-validation to minimize mean-squared-error loss, while preventing overfitting through regularization and data normalization.

The ML framework treats each pose component: three rotational (R_x_, R_z_, R_z_) and three translational (T_x_, T_y_, T_z_) as a separate regression head, ensuring modularity and allowing comparative analysis across algorithms. Training and evaluation were conducted on stratified subsets to preserve workspace diversity, and performance metrics, including Mean Absolute Error (MAE), Root Mean Squared Error (RMSE), and Coefficient of Determination (*R* ^2^), were computed for quantitative benchmarking. By leveraging these complementary learning paradigms, the framework establishes a data-driven surrogate capable of accurately reproducing the nonlinear kinematic behavior of the C-arm with surgical table system, thereby enabling efficient prediction and analysis across complex surgical configurations.

### 3.5 Machine learning model architectures

This paper assesses five supervised machine learning models to predict collision-free C-arm end-effector pose from joint configurations inputs and *vice versa*. The selected models span linear, instance-based, ensemble-based, and deep learning paradigms, enabling a comprehensive comparison of predictive performance under varying degrees of model complexity. The architectural details and configuration strategies for each model are described in the following subsections.

#### 3.5.1 Ridge regression

Ridge Regression serves as a baseline linear model that incorporates L2 regularization to mitigate overfitting and enhance numerical stability. Unlike standard Linear Regression, which may suffer from high variance in the presence of multicollinearity or high-dimensional feature spaces, Ridge Regression penalizes large coefficient values, thus reducing model sensitivity to noise and correlated input features. The model minimizes the residual sum of squares while applying a penalty on the magnitude of regression coefficients. The regularization strength α , a critical hyperparameter controlling the degree of shrinkage, was optimized using 5-fold cross-validation. This regularization is particularly beneficial in scenarios where joint variables (e.g., multiple joint movements of C-arm and operating table) exhibit interdependence, as it prevents overfitting and improves generalization to unseen poses. Ridge Regression was selected over standard Linear Regression for its improved robustness and ability to handle ill-conditioned or collinear data, both of which are common in robotic systems involving multiple coupled degrees of freedom. Its efficiency, simplicity, and interpretability make it a suitable candidate for benchmarking against more complex models.

#### 3.5.2 Gradient boosting machines

Gradient Boosting Machine (GBM) builds a sequence of weak learners typically shallow decision trees where each new learner attempts to correct the residual errors of the ensemble’s cumulative prediction. Unlike Random Forests, GBM relies on additive model building and is sensitive to overfitting, necessitating careful regularization. Core hyperparameter, the learning rate all of which has been optimized through cross-validation. GBM is particularly adept at capturing complex, non-linear relationships in structured datasets, making it well-suited for learning the mapping between high-dimensional pose inputs and corresponding joint configurations.

**FIGURE 4:**
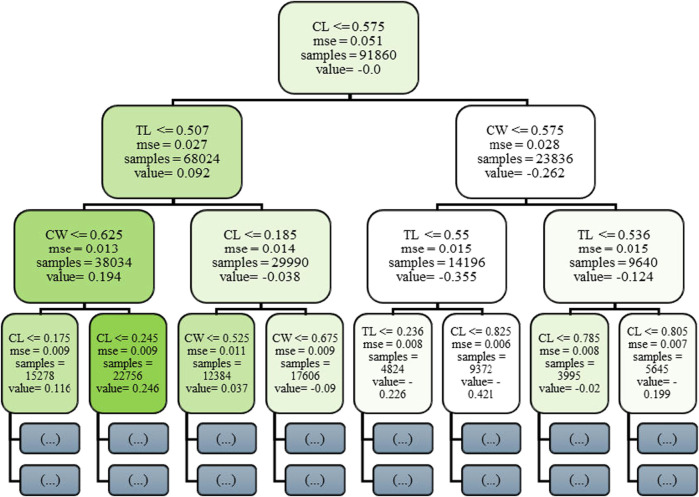
Visualization of the initial decision tree (Stage 0) in the Gradient Boosting Machine (GBM) model for C-arm pose. Prediction.

Figure 4 presents the initial decision tree (Stage 0) in the Gradient Boosting Machine ensemble, offering critical insight into the model’s foundational learning dynamics. GBM constructs an additive sequence of weak learners typically decision trees each aimed at correcting the residual errors of its predecessors. This first tree is particularly informative, as it reveals how the model initiates the mapping between joint-space input features and predicted C-arm pose components. Internal nodes within the tree represent binary decision rules based on input features such as C-arm Wigwag (CW), Table Longitudinal (TL) etc., with each node applying a threshold criterion to partition the dataset. Depending on whether an input value satisfies the threshold condition, the instance is routed to the left or right child node. Terminal nodes, or leaves, output scalar prediction values that contribute incrementally to the model’s final prediction through boosting. While the complete GBM comprises hundreds of such trees, this single-tree visualization facilitates interpretability by highlighting early feature selection, decision thresholds, and hierarchical rule formation attributes that are vital for clinical transparency and trust in automated pose estimation systems.

#### 3.5.3 K-Nearest Neighbors

K-Nearest Neighbors (KNN) is a non-parametric, instance-based learning algorithm that infers the output of a test sample by aggregating the labels of its *k* closest neighbors in the feature space. The model operates under the assumption that similar inputs yield similar outputs. The optimal value of *k*, along with the choice of distance metric and weighting scheme, has been selected through cross-validation. To improve robustness, a distance-weighted voting mechanism is employed, allowing closer neighbors to contribute more significantly to the final prediction. KNN provides a simple yet powerful baseline, especially in data-rich environments.

#### 3.5.4 Random Forest

Random Forest (RF) is an ensemble learning algorithm that constructs multiple decision trees using bootstrap aggregation (bagging). Each tree is trained on a randomly sampled subset of the training data and a random subset of features, promoting model diversity. Predictions are made by averaging the outputs across all trees, reducing variance and improving generalization. Key hyperparameter, the number of trees (estimators) has been tuned using cross-validation. This model is particularly effective in capturing non-linear dependencies and handling feature interactions without requiring extensive preprocessing.

**FIGURE 5:**
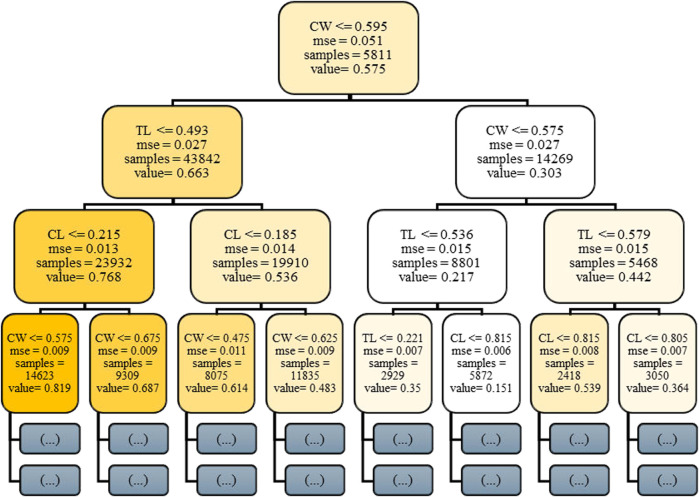
Visualization of the first decision tree (depth truncated to 3) from the trained Random Forest model.

To elucidate the internal decision-making process of the ensemble, Figure 5 depicts a truncated representation of the first decision tree extracted from the trained Random Forest regression model. The visualization is deliberately constrained to a depth of three hierarchical levels to preserve interpretive clarity while exposing the fundamental feature-partitioning logic. Each internal node encodes a threshold-driven decision rule applied to a specific joint-space parameter, for example, C-arm Wigwag (CW) or Table Longitudinal displacement (TL) that iteratively bisects the data into increasingly homogeneous subregions. Terminal (leaf) nodes summarize the mean predicted output value, the corresponding mean squared error (MSE), and the sample cardinality within each partition. Prior to training, all input variables were normalized to the [0, 1] interval; consequently, the threshold values displayed represent normalized magnitudes, while their physical analogues correspond to degree for revolute joints and mm for prismatic joints. This interpretive rendering provides a concise, yet transparent exposition of how the Random Forest architecture hierarchically integrates multi-joint dependencies to approximate the nonlinear mapping between the modular C-arm system configuration and its resultant Cartesian pose components.

#### 3.5.5 Deep Neural Network

The DNN was explored in three progressively optimized configurations to evaluate its effectiveness in learning non-linear mappings between pose parameters and joint configurations. Each architecture was implemented as a feedforward network composed of fully connected layers and Rectified Linear Unit activation functions, with a six-neuron output layer corresponding to the 6D pose vector (R_x_, R_y_, R_z_, T_x_, T_y_, T_z_).

The initial DNN model adopted a lightweight architecture with three hidden layers of 128, 64, and 32 neurons, respectively, followed by the output layer. Although this configuration offered computational efficiency and acceptable baseline performance, it exhibited limited capacity in modeling complex, high-dimensional input relationships, often leading to underfitting.

**FIGURE 6:**
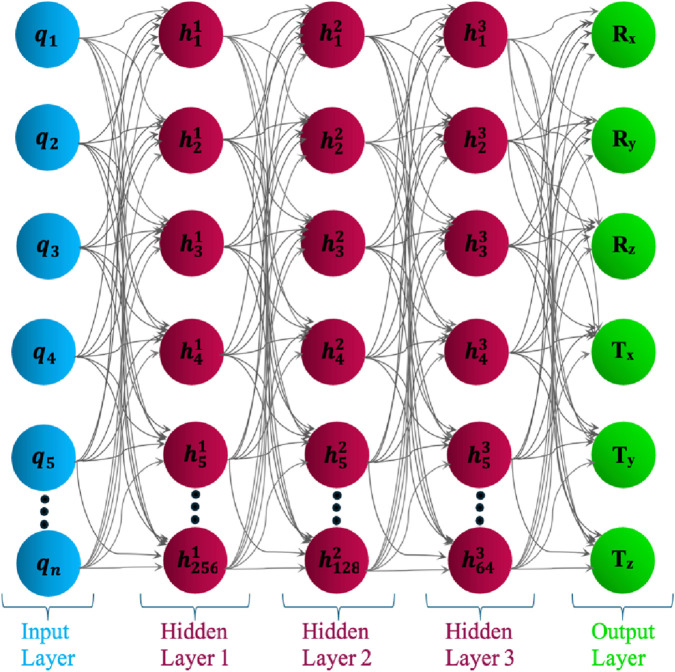
Architecture of the Deep Neural Network model with variable joint input layer and fixed 6D pose output.

To enhance the learning capability of the network, the architecture has been expanded to include three larger hidden layers with 256, 128, and 64 neurons, respectively. This deeper configuration as shown in Figure 6, significantly improved performance by enabling the network to learn hierarchical abstractions from the input data. Dropout layers with rates of 0.3 and 0.2 were inserted after the first and second hidden layers to reduce overfitting and improve generalization.

The architecture of a DNN has been designed to map joint space inputs to corresponding 6D pose outputs. The input layer consists of 5–nine neurons, each representing a joint variable $q_{i}$ , where the number of inputs depends on the modular configuration of the integrated C-arm system. The output layer comprises six neurons, each corresponding to one component of the predicted pose: R_x_, R_y_, R_z_ (rotational angles) and T_x_, T_y_, T_z_ (translational positions). These six outputs together form the full 6D pose vector that defines the end-effector configuration of the robotic system in task space.

In the final variant, the same deep architecture (256/128/64) has been retained, but the input features were first processed using PCA. PCA served as a dimensionality reduction technique to project the original feature space into a lower-dimensional, uncorrelated subspace, preserving the most significant components. This not only improved training efficiency but also reduced model complexity and enhanced robustness against multicollinearity in the input space.

All DNN models were trained using the Adam optimizer with a learning rate of 0.001 and MSE as the loss function. To stabilize convergence and prevent overfitting, early stopping with a patience of 30 epochs was applied. A dynamic learning rate schedule was also employed, reducing the learning rate by 10% if no improvement was observed in validation loss for 20 consecutive epochs.

The final DNN configuration with PCA preprocessing and a deeper architecture demonstrated superior performance in capturing the complex, non-linear relationships necessary for accurate and collision-free pose estimation in a high-dimensional modular C-arm workspace.

### 3.6 System configuration and training methodology

The training and evaluation of models were performed on a high-performance workstation configured with a 13th Gen Intel® Core™ i7-13700KF processor (3.4 GHz, 16 physical cores, 24 logical threads) and 16 GB of DDR4 RAM, running a 64-bit Windows 11 operating system. The system was equipped with an NVIDIA GeForce RTX 4070 GPU featuring 12GB GDDR6X memory and full support for CUDA and cuDNN acceleration. Model development was conducted using Python 3.12.3, with essential scientific and machine learning libraries including NumPy 1.26.4, pandas 2.2.2, scikit-learn 1.4.2, and SciPy 1.13.3. Development and experimentation were carried out within JupyterLab 4.0.11, offering an interactive environment for data preprocessing, model training, and result visualization. To ensure reproducibility and effective dependency management, all experiments were executed within a dedicated Conda virtual environment, encapsulating the necessary packages and configurations.

To optimize model performance and mitigate overfitting, hyperparameter tuning was systematically conducted for all five machine learning models using cross-validation. Each model was evaluated over a predefined, algorithm-specific hyperparameter search space. The DNN model was designed with four fully connected layers arranged sequentially, comprising 256, 128, 64, and output neurons, respectively. Dropout layers with rates of 0.3 and 0.2 were interleaved between the dense layers to provide regularization and mitigate overfitting, resulting in a total of six hidden layers in the architecture. For tree-based models such as Random Forest and Gradient Boosting Machines, key hyperparameters including the number of decision trees, and learning rate were tuned extensively. The KNN model was configured by testing different values for the number of neighbors and selecting the one that yielded the lowest validation error. As for the Ridge Regression model, regularization strength (α) was varied to determine the optimal balance between bias and variance.

To prevent overfitting and ensure generalization, the dataset was consistently partitioned into training (70%), validation (15%), and testing (15%) subsets across all five machine learning models. Early stopping was applied during DNN training, with a patience of 30 epochs based on validation loss. In addition, a dynamic learning rate scheduler was employed, reducing the learning rate by 10% if no improvement was observed in validation loss for 20 consecutive epochs. This approach helped escape local minima and contributed to further optimization of the model, as evidenced by the consistent decline in validation error beyond epoch 120. Table 5 summarizes the optimized hyperparameters employed across all machine learning models and Degrees of Freedom, distinguishing between rotational (R_x_, R_y_, R_z_) and translational (T_x_, T_y_, T_z_) components of the 6D pose. These parameters are systematically selected using cross-validation to maximize generalization and prediction accuracy while avoiding overfitting, particularly in high-dimensional input spaces. For Ridge Regression, the regularization strength (α) controls the trade-off between model complexity and stability. A value of 1.0 is consistently assigned to rotational components in higher DoF settings (e.g., 9DoF and 8DoF) to suppress overfitting due to angular coupling. In contrast, lower values (as small as 0.01) are allocated to translational components to retain sensitivity to small positional shifts. In the case of GBM, the learning rate directly affects convergence speed and model granularity. Higher rates (0.2) are assigned to most pose components to ensure rapid learning, except for rotational components in 9DoF where lower rates (0.1) improve stability in complex angular mappings. The learning rate is tuned more conservatively in configurations with higher rotational variance. For KNN, the number of neighbors (k) significantly impacts the model’s local sensitivity. A larger k (e.g., 9) is generally preferred for translational prediction to smooth noise in high DoF settings, while smaller values (3–5) are applied to rotational DoFs, where fine-grained prediction benefits from localized context. The RF model depends on the number of decision trees for ensemble robustness. Larger ensembles (e.g., 300 trees) are used for translational outputs across 8DoF and 9DoF systems to manage spatial complexity. For rotational components or lower DoF models, fewer trees (100–200) suffice, maintaining computational efficiency without degrading performance. The DNN architecture remains consistent across all configurations, comprising three fully connected layers (256, 128, 64 neurons) with dropout rates of 0.3 and 0.2 for regularization. The batch size is fixed at 32, and training proceeds for up to 300 epochs with early stopping based on validation loss. A learning rate scheduler reduces the learning rate by 10% after 20 stagnant epochs, facilitating convergence and avoiding local minima. This structured tuning approach ensures that each model adapts to the complexity and variability introduced by different DoF configurations, enhancing predictive accuracy and robustness across translational and rotational subspaces.

### 3.7 Evaluation protocol and generalization strategy

To ensure reproducibility, statistical independence, and the elimination of data leakage, all machine-learning models were evaluated using a rigorously isolated data-handling protocol. For each DoF configuration, the complete dataset was partitioned into 70% training, 15% validation, and 15% testing subsets using stratified random sampling, thereby preserving the spatial and kinematic diversity of the modular C-arm workspace. Each split was generated with a fixed random seed to guarantee deterministic reproducibility, while ensuring that no individual pose or configuration appeared in more than one subset.

All numerical features were normalized using the StandardScaler implementation in *scikit-learn*, applying a z-score transformation defined as Equation 1 as:

$$x^{\prime }=\frac{x-\mu_{train}}{\sigma_{train}}$$ (1)

where $\mu_{train}$ and $\sigma_{train}$ denote the mean and standard deviation computed exclusively from the training subset. This approach maintains statistical independence between subsets and eliminates information leakage, a subtle yet common source of optimistic bias in regression tasks. Standardizing within the training domain also harmonizes feature magnitudes across translational and rotational dimensions, thereby improving numerical conditioning, gradient stability for deep models, and feature weighting consistency for distance-based algorithms such as KNN. Model evaluation followed a multi-stage validation pipeline designed to capture both local and global generalization behavior. During the development phase, hyperparameters were optimized through k-fold cross-validation within the training and validation sets. The finalized models were retrained using the merged training and validation data and evaluated on the independent test subset to derive an unbiased estimate of generalization performance.

To examine spatial generalization beyond statistical sampling, an additional domain-level validation step was conducted using unseen workspace segments pose clusters intentionally withheld during training. This analysis confirmed that predictive consistency extended to spatially distinct regions of the workspace, demonstrating that the learned mappings captured functional relationships rather than memorized local geometries.

The top-performing Gradient Boosting Machine (GBM) model further underwent a robustness assessment under controlled perturbations to emulate encoder drift, sensor quantization, and minor mechanical backlash. Gaussian noise with zero mean and standard deviations of σ=0.01,0.05,0,10 was injected into the test inputs, and variations in MAE, RMSE, and *R* ^2^ were analyzed to verify the GBM’s numerical resilience and stability under noise (see Section 4.C).

Conclusively, model performance was quantified independently for the training, validation, and test subsets using MAE, RMSE, and the *R* ^2^. This hierarchical evaluation framework integrating isolated feature scaling, stratified partitioning, cross-domain validation, and noise-based robustness testing ensures that all reported results are statistically sound, free from normalization or sampling leakage, and demonstrably generalizable across the complete 9-DoF modular C-arm workspace.

### 3.8 Evaluation metrics

To quantitatively evaluate the performance of the machine learning models in predicting collision-free poses of modular C-arm system, four widely accepted regression performance metrics: Mean Absolute Error (MAE), Mean Squared Error (MSE), Root Mean Squared Error (RMSE), and the Coefficient of Determination (*R* ^2^) are employed. These metrics provide complementary perspectives on model accuracy, bias, and variance, and have been validated across numerous benchmarking studies in regression modeling (Sekeroglu et al., 2022; Botchkarev, 2018). Before presenting these metrics, the notation used in the equations is depicted as follows:

○ $y_{i}$ : The true value (ground truth) of the $i^{\text{th}}$ data point.
○ ${\hat{y}}_{i}$ : The predicted value output by the model for the $i^{\text{th}}$ sample.
○ $\bar{y}$ : The mean of the true values across all samples.
○ n : The total number of data samples used for evaluation.

#### 3.8.1 Mean absolute error (MAE)

MAE quantifies the average absolute difference between the predicted and actual values. It is particularly useful for understanding the magnitude of errors in physical units (e.g., millimeters or degrees) and is robust to outliers compared to squared-error metrics. Lower MAE values indicate higher predictive accuracy. It is calculated using the Equation 2 as follows:

$$\text{MAE}=\frac{1}{n}\sum_{i=1}^{n}\left|y_{i}-{\hat{y}}_{i}\right|$$ (2)

MAE is particularly suitable for applications where the cost of prediction errors grows linearly and is less sensitive to outliers compared to MSE (Botchkarev, 2018).

#### 3.8.2 Mean squared error (MSE)

MSE calculates the average of the squared differences between true and predicted values. It penalizes larger errors more heavily, making it useful for highlighting model deviations with high impact. It is calculated using Equation 3 as follows:

$$\text{MSE}=\frac{1}{n}\sum_{i=1}^{n}{\left(y_{i}-{\hat{y}}_{i}\right)}^{2}$$ (3)

This metric is widely used for its strong mathematical properties in optimization and model training (Dumre et al., 2024).

#### 3.8.3 Root mean squared error (RMSE)

RMSE is the square root of MSE and retains the original unit of measurement, making it suitable for direct interpretability in physical domains. It is calculated using Equation 4 as follows:

$$\text{RMSE}=\sqrt{\frac{1}{n}\sum_{i=1}^{n}{\left(y_{i}-{\hat{y}}_{i}\right)}^{2}}$$ (4)

The sensitivity of RMSE to larger errors can be both a strength and a drawback, depending on application context (Dumre et al., 2024).

#### 3.8.4 Coefficient of determination (*R* ^2^)

*R* ^2^, known as the coefficient of determination, quantifies the proportion of variance in the dependent (output) variable that can be explained by the independent (input) variables. It serves as a statistical measure of a model’s goodness of fit (Rhinehart, 2016). The coefficient of determination or *R* ^2^ is shown in Equation 5 as (Rhinehart, 2016):

$$R^{2}=1-\frac{{\text{SSD}}_{m}}{{\text{SSD}}_{y}}=1-\frac{\sum_{i=1}^{n}{\left(y_{i}-{\hat{y}}_{i}\right)}^{2}}{\sum_{i=1}^{n}{\left(y_{i}-\bar{y}\right)}^{2}}$$ (5)

where SSD_y_ denotes the sum of squared deviations from the mean of the dependent variable (i.e., the total variance in the response data), and SSD_m_ represents the sum of squared deviations of the model residuals (i.e., prediction errors). An *R* ^2^ value close to 1 indicates that the model predictions align closely with the true values, reflecting high predictive accuracy. Conversely, a value near 0 suggests that the model fails to capture the underlying patterns in the data (Sekeroglu et al., 2022).

These metrics were computed for three rotational components (R_x_, R_y_, R_z_) and three translational components (T_x_, T_y_, T_z_), which together constitute the complete 6D pose of the C-arm system within the clinical workspace. Evaluation was carried out across training, validation, and testing subsets for all five machine learning models. In the context of medical robotics and intraoperative imaging, precise task-space prediction is essential to ensure accurate end-effector positioning while maintaining patient safety. Accurate estimation of both position and orientation directly impacts the system’s ability to achieve collision-free pose, particularly in constrained surgical environments. This metric-based evaluation supports a rigorous comparison of model performance in terms of pose estimation accuracy, consistency across data splits, and generalization to unseen configurations all of which are critical for deploying intelligent robotic systems that enhance imaging access without compromising clinical safety.

## 4 Results and discussion

### 4.1 Machine learning-based forward kinematics

**FIGURE 7:**
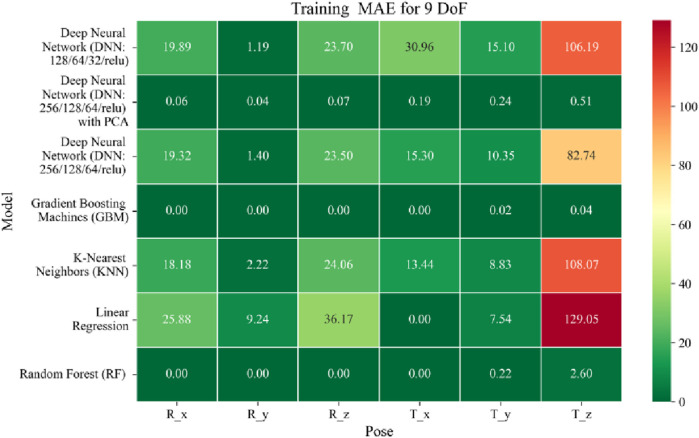
Heatmap of training set Mean Absolute Error for machine learning models.

**FIGURE 8:**
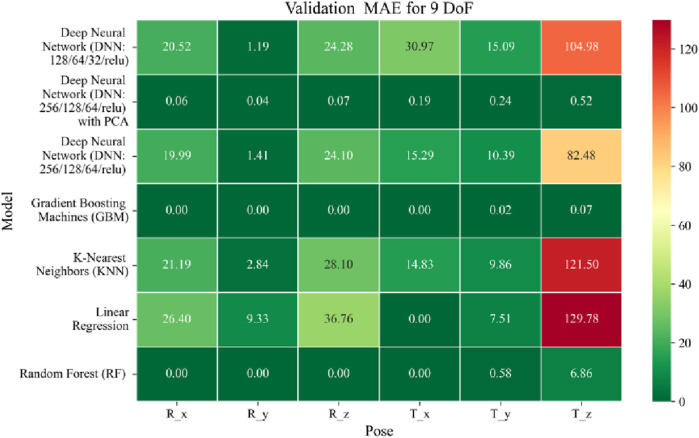
Heatmap of validation set Mean Absolute Error for machine learning models.

**FIGURE 9:**
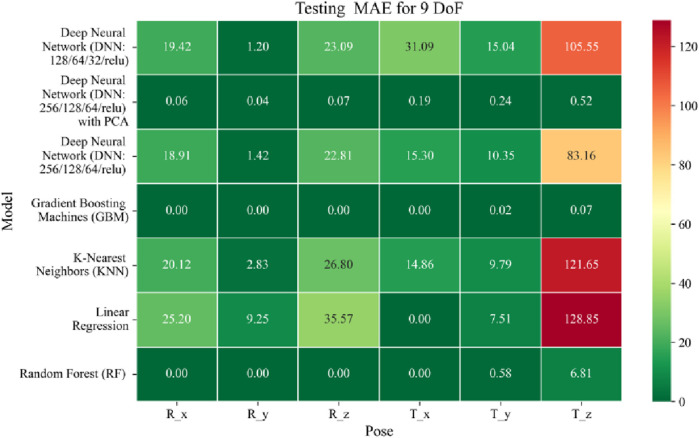
Heatmap of test set Mean Absolute Error for machine learning models.

**FIGURE 10:**
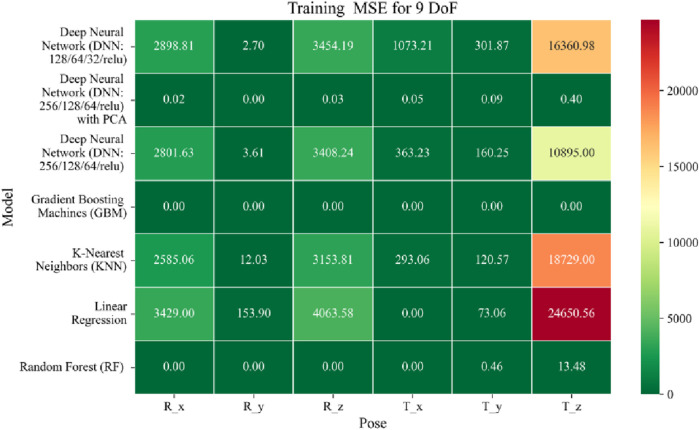
Heatmap of training set Mean Squared Error for machine learning models.

**FIGURE 11:**
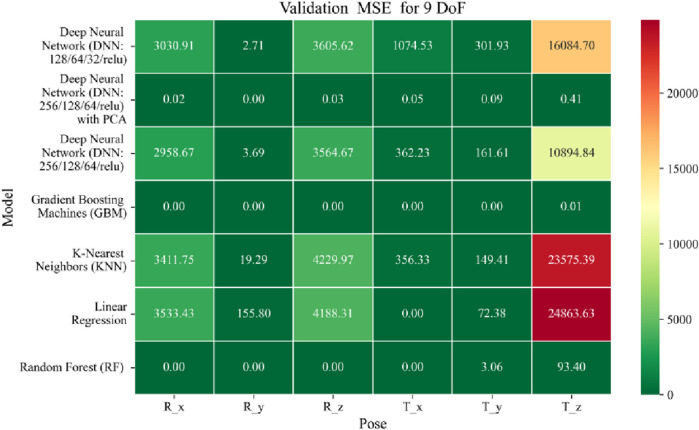
Heatmap of validation set Mean Squared Error for machine learning models.

**FIGURE 12:**
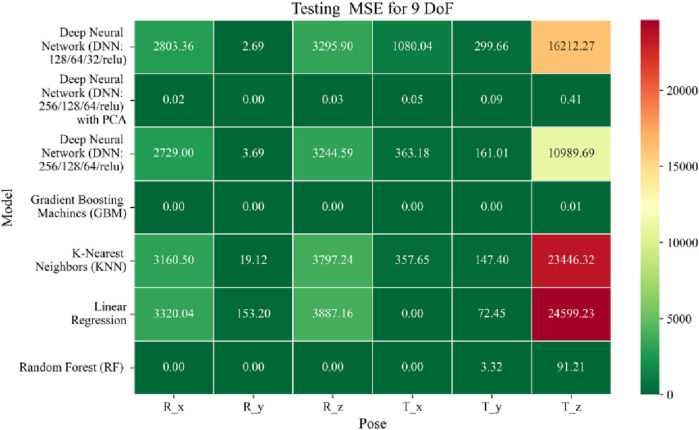
Heatmap of test set Mean Squared Error for machine learning models.

**FIGURE 13:**
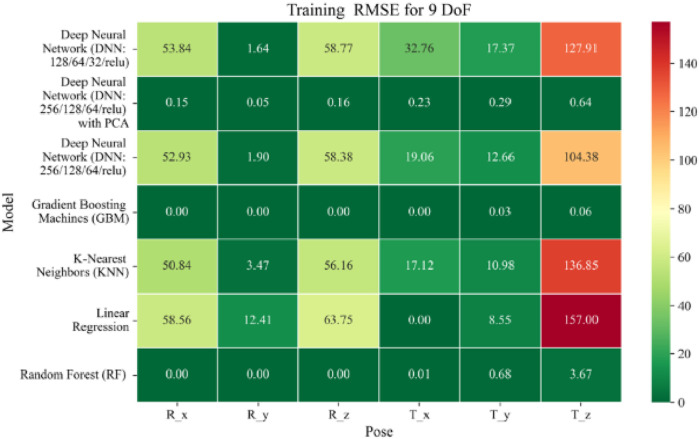
Heatmap of training set Root Mean Squared Error for machine learning models.

**FIGURE 14:**
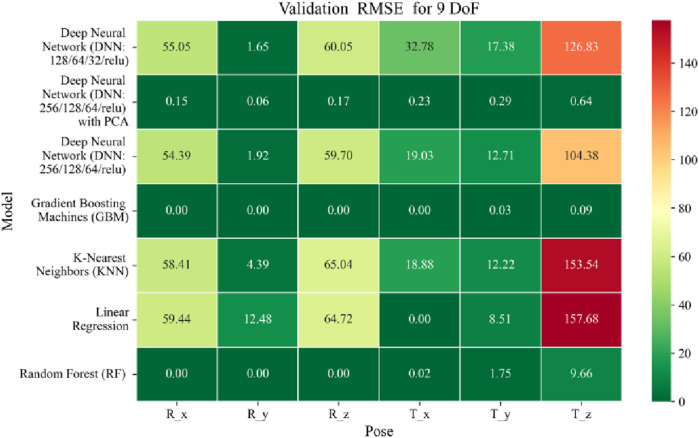
Heatmap of validation set Root Mean Squared for machine learning models.

**FIGURE 15:**
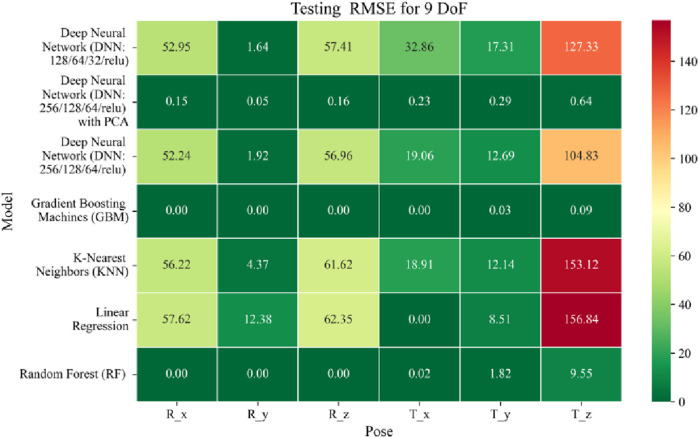
Heatmap of test set Root Mean Squared Error for machine learning models.

**FIGURE 16:**
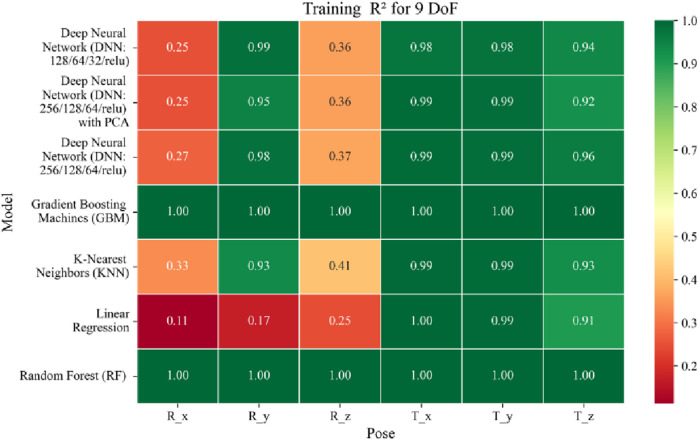
Heatmap of training set Coefficient of Determination for machine learning models.

**FIGURE 17:**
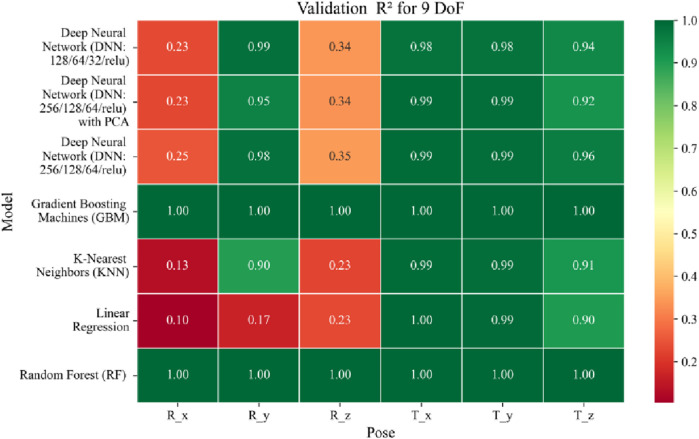
Heatmap of validation set Coefficient of Determination for machine learning models.

**FIGURE 18:**
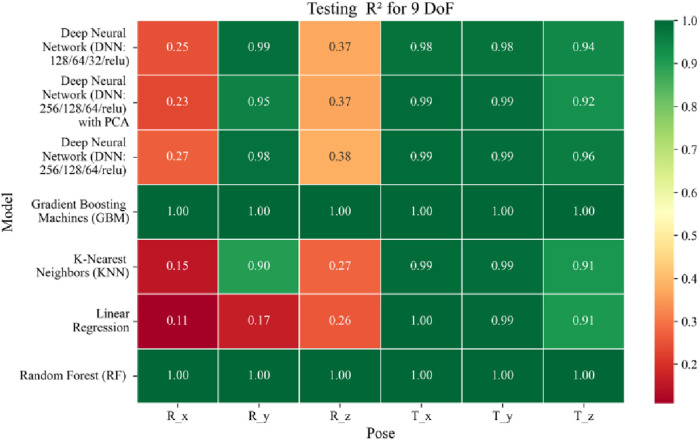
Heatmap of test set Coefficient of Determination for machine learning models.

The application of machine learning models is investigated to solve the forward kinematics problem, wherein joint variables from the 9DoF C-arm system serve as inputs to predict the corresponding 6D end-effector poses comprising three Euler angles and three translational components. A range of regression techniques, including linear models and ensemble-based approaches, is employed to learn the complex mapping from joint space to task space. To evaluate model performance comprehensively and support the selection of optimal learning strategies for high-dimensional pose estimation, twelve heatmaps are generated as shown in Figures 7–18. These visualizations report standard regression metrics described in the last section across the training, validation, and testing phases. These visualizations compare model accuracy for each of the six pose components: three translation vectors (t_x_, t_y_, t_z_) and three Euler angles (r_x_, r_y_, r_z_) under the 9DoF configuration which are represented in the heatmaps as T_x, T_y, T_z and R_x, R_y, R_z respectively. For clarity, all errors in translational vectors are reported in millimeters (mm), while errors in Euler angles are expressed in degrees (°).

Among all models as represented in Figures 7–9, GBM demonstrate the lowest MAE values. For Euler angles, the training, validation, and testing MAEs are all zero, indicating perfect alignment between predicted and ground truth rotations. For translational vectors, GBM achieves training MAEs ranging from 0 to 0.04 mm, validation MAEs from 0 to 0.07 mm, and testing MAEs from 0 to 0.07 mm. The DNN with PCA performs nearly as well. For Euler angles, MAEs range from 0° to 0.07° across all three phases. For translational components, training MAE ranges from 0.19 to 0.51 mm, validation MAE from 0.19 to 0.52 mm, and testing MAE from 0.19 to 0.52 mm. The standard DNN without PCA shows higher error magnitudes. For Euler angles, training MAEs range from 1.40° to 23.50°, validation MAEs from 1.41° to 24.10°, and testing MAEs from 1.42° to 22.81°. For translations, training MAEs range from 10.35 to 82.74 mm, validation from 10.39 to 82.48 mm, and testing from 10.35 to 83.16 mm.

The MSE analysis, as illustrated in Figures 10–12, provides insight into the magnitude of squared prediction errors across models and datasets, while the RMSE analysis in Figures 13–15 reaffirms MAE trends by offering a scale-sensitive interpretation of model performance. GBM again performs exceptionally well, with 0° RMSE for Euler angles across all splits and translational RMSE ranging from 0 to 0.06 mm (training), 0–0.09 mm (validation), and 0–0.09 mm (testing). For DNN with PCA, Euler angle RMSE spans 0.05°–0.16° (training), 0.06°–0.17° (validation), and 0.05°–0.16° (testing). Translational RMSE lies between 0.23 and 0.64 mm consistently across all three phases. The DNN without PCA yields RMSE values for Euler angles between 1.90° and 58.38° (training), 1.92°–59.70° (validation), and 1.92°–56.96° (testing). For translational vectors, RMSE spans 12.66–104.38 mm (training), 12.71–104.38 mm (validation), and 12.69–104.83 mm (testing).

The coefficient of determination (*R* ^2^) as depicted in Figures 16–18 provides a normalized measure of how well the predicted values explain the variance in the actual pose components. Among all models evaluated, GBM achieves a perfect *R* ^2^ of 1.00 across all pose components encompassing both Euler angles and translational vectors during training, validation, and testing. While this indicates that the model captures the full variance present in the target data, such ideal scores, particularly when accompanied by near-zero MAE and RMSE values, require cautious interpretation. To address potential concerns related to overfitting or data leakage, the evaluation protocol includes a clearly defined train-test split strategy and *k*-fold cross-validation during model development and hyperparameter tuning. These procedures ensure that model performance is not attributed to memorization or overlap across datasets. Nevertheless, validation on independent datasets remains essential to further establish the model’s generalizability and robustness in real-world applications. The Deep Neural Network with PCA demonstrates high *R* ^2^ values for translational components across all splits, ranging from 0.92 to 0.99, indicating that the model captures nearly all variance in the positional data. This is consistent with the low RMSE values and reflects the relative linearity and predictability of translational motion from joint inputs. For rotational components, however, the *R* ^2^ values are more modest ranging from 0.25 to 0.36 (training), 0.23 to 0.35 (validation), and 0.23 to 0.37 (testing). These lower scores are not necessarily indicative of poor performance; rather, they reflect the inherently higher complexity and sensitivity of predicting angular components (Euler angles), where even small discrepancies can result in a large proportion of unexplained variance. Additionally, rotational motion tends to be more nonlinear and sensitive to joint interactions, making it harder for the model to explain variance compared to translational motion. The standard DNN without PCA follows a similar trend, achieving high *R* ^2^ values for translational vectors ranging from 0.96 to 0.99, and lower *R* ^2^ values for rotational components ranging from 0.25 to 0.35 across all three datasets. The slightly lower *R* ^2^ scores compared to the PCA-enhanced version suggest that feature dimensionality reduction plays a role in improving angular variance modeling by reducing noise and multicollinearity in the input space.

In summary, GBM establishes itself as the most accurate and reliable forward kinematics model for the 9DoF modular C-arm system, achieving near-zero error metrics and perfect variance capture across all pose components. The DNN with PCA emerges as a viable alternative, particularly for translational tasks, while the standard DNN highlights the challenges posed by high-dimensional, nonlinear pose mapping.

### 4.2 Machine learning-based inverse kinematics

**FIGURE 19:**
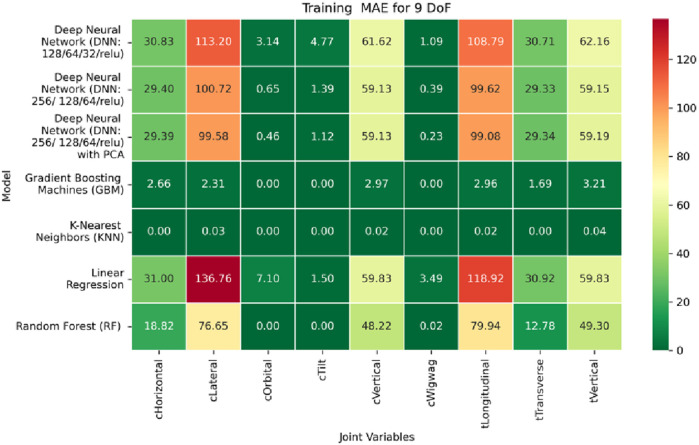
Heatmap of training set Mean Absolute Error for Inverse Kinematics ML.

**FIGURE 20:**
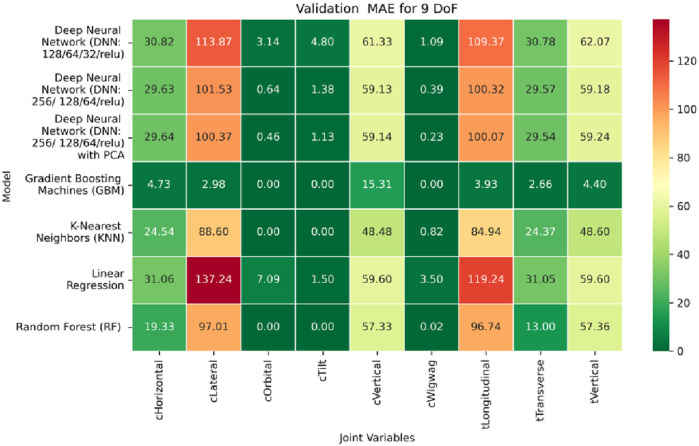
Heatmap of validation set Mean Absolute Error for Inverse Kinematics ML.

**FIGURE 21:**
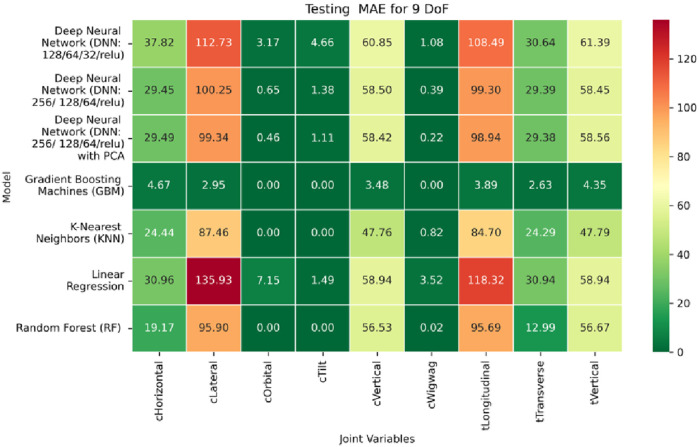
Heatmap of test set Mean Absolute Error for Inverse Kinematics ML.

**FIGURE 22:**
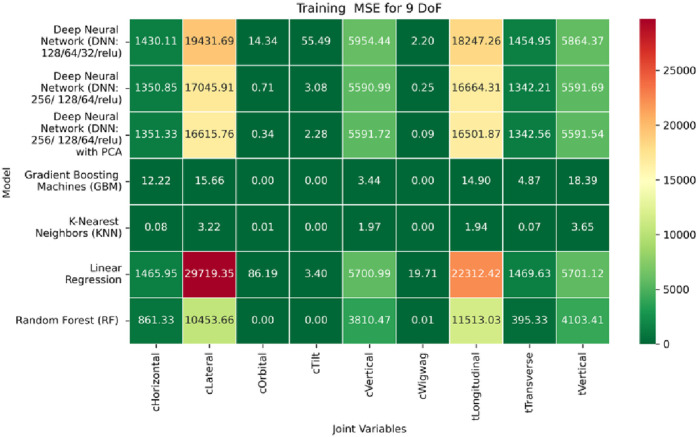
Heatmap of training set. Mean Squared Error for inverse kinematics ML.

**FIGURE 23:**
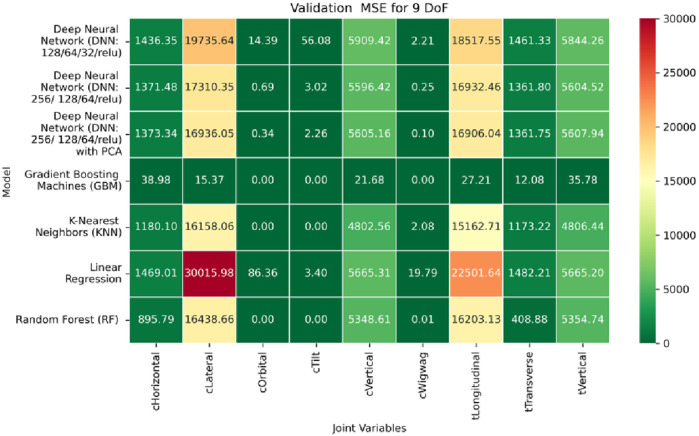
Heatmap of validation set. Mean Squared Error for inverse kinematics ML.

**FIGURE 24:**
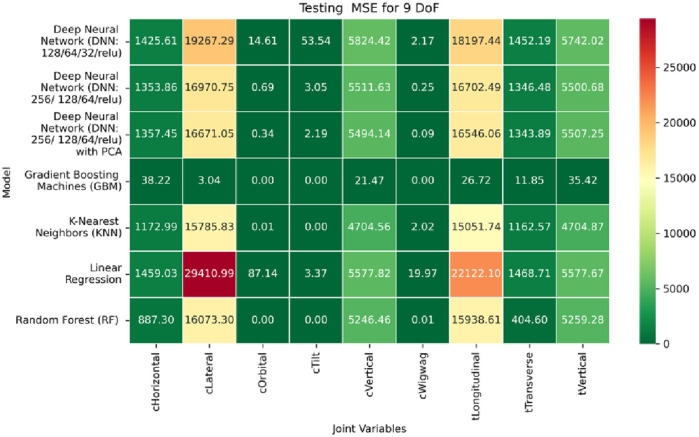
Heatmap of test set. Mean Squared Error for inverse kinematics ML.

**FIGURE 25:**
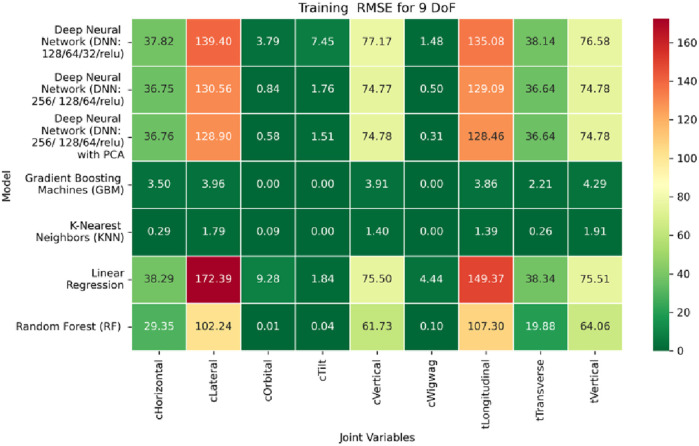
Heatmap of training set. Root Mean Squared Error for inverse kinematics ML.

**FIGURE 26:**
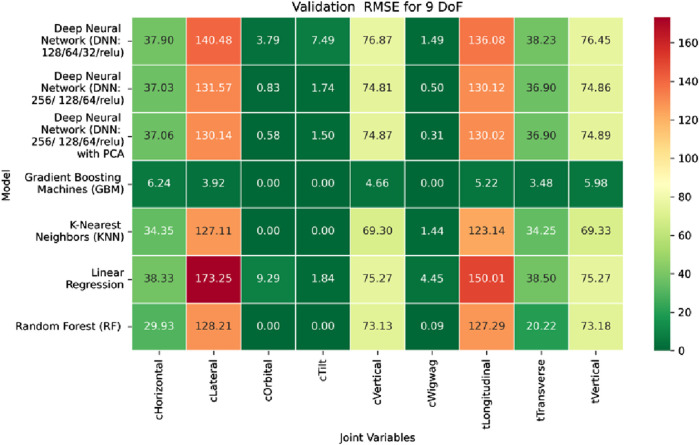
Heatmap of validation set. Root Mean Squared Error for inverse kinematics ML.

**FIGURE 27:**
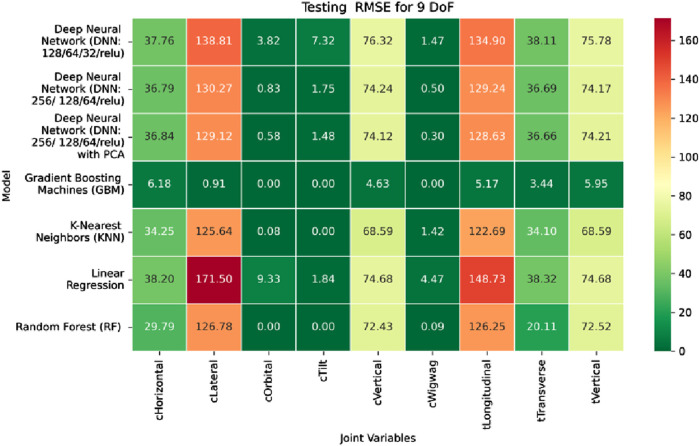
Heatmap of test set. Root Mean Squared error for inverse kinematics ML.

**FIGURE 28:**
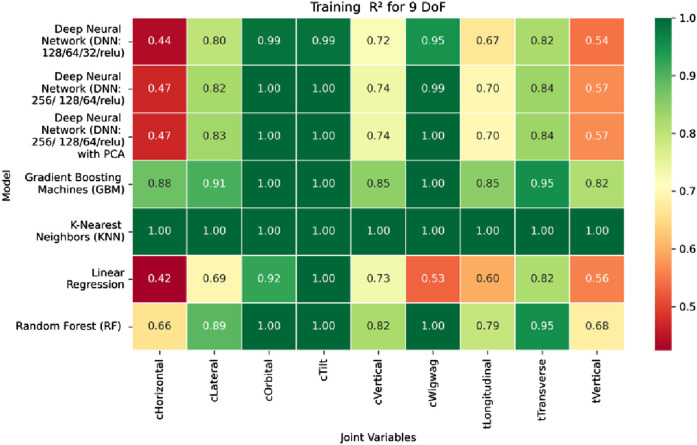
Heatmap of training set. coefficient of determination for inverse kinematics ML.

**FIGURE 29:**
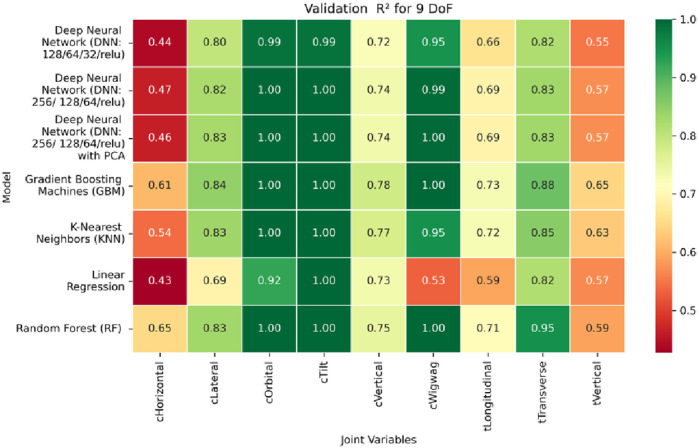
Heatmap of validation set. coefficient of determination for inverse kinematics ML.

**FIGURE 30:**
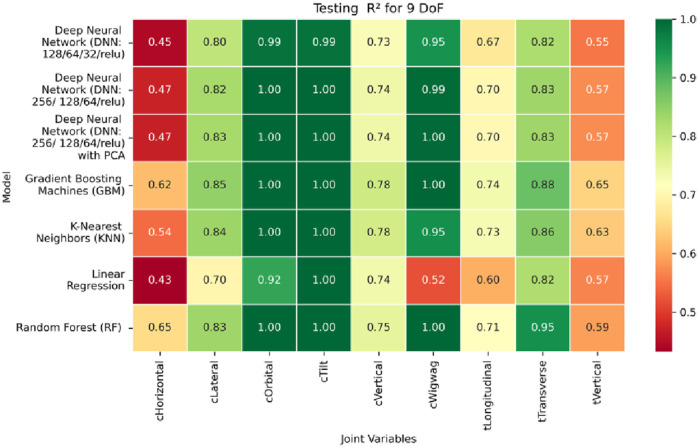
Heatmap of test set. coefficient of determination for inverse kinematics ML.

To address the inverse kinematics problem in a 9DOF C-arm robotic system with operating table, we recall that the input features consist of translation vectors (t_x_, t_y_, t_z_) and rotational components encoded as Euler angles (r_x_, r_y_, r_z_). The output space encompasses six C-arm joints and three operating table joints. Similarly, machine learning models are evaluated against the evaluation metrics, and their performance are manifested through twelve heatmaps (Figures 19–30) across training, validation, and test datasets.

Among all evaluated approaches, the GBM model consistently delivers superior accuracy and generalization. For revolute joints specifically the C-arm Wigwag, Tilt, and Orbital joints GBM achieves perfect predictive accuracy, attaining MAE and RMSE values of 0.00° across all phases. This remarkable performance demonstrates the model’s capacity to effectively learn the nonlinear transformation from Cartesian rotational descriptors to joint angles, despite the high dimensionality and inherent complexity of the rotational kinematics involved.

In contrast, performance on prismatic joints exhibits variation influenced by joint-specific mechanical constraints and pose-to-configuration mapping complexity. For the C-arm Vertical, C-arm Horizontal, Table Vertical, Table Longitudinal, and Table Transverse axes, error magnitudes differ across datasets. During the training phase, as illustrated in Figure 19, the Table Transverse joint records the lowest MAE at 1.69 mm, while the Table Vertical joint exhibits the highest MAE at 3.21 mm. In the validation phase, shown in Figure 20, the C-arm Horizontal joint incurs the largest error at 4.73 mm, whereas Table Transverse remains the most accurately predicted with an MAE of 2.66 mm. A similar trend is observed during testing, as shown in Figure 21, where MAE values range from 2.63 mm (Table Transverse) to 4.67 mm (C-arm Horizontal).

The RMSE results further reinforce these trends, providing deeper insight into the distribution and scale of prediction errors. While Figures 22–24 present the MSE distributions across training, validation, and testing phases, the corresponding RMSE outcomes are illustrated in Figures 25–27. For prismatic joints, training RMSE values span from 2.21 mm (Table Transverse) to 4.29 mm (Table Vertical). In the validation set, RMSE increases modestly, ranging from 3.48 mm to 6.24 mm, with the C-arm Horizontal joint again showing the highest deviation. Testing RMSE remains within acceptable bounds, varying between 3.44 mm and 6.18 mm. Notably, all translational errors fall within clinically acceptable thresholds, underscoring the model’s applicability for high-precision medical imaging and surgical positioning tasks.

Regarding variance explanation, the coefficient of determination results, as depicted in Figures 28–30, further validate the predictive robustness of the GBM model. As shown in Figure 28, GBM achieves an *R* ^2^ score of 1.00 for all rotational joints in the training phase, affirming its perfect fit across all angular components. For translational joints, training *R* ^2^ values range from 0.88 (C-arm Horizontal) to 0.99 (Table Transverse). In the validation phase, illustrated in Figure 29, a moderate decline in predictive performance is observed, with *R* ^2^ values spanning 0.61 to 0.88. The testing phase results presented in Figure 30 show a similar distribution, reinforcing the model’s consistency across unseen data. Notably, the C-arm Horizontal joint characterized by an extensive motion envelope and nonlinear inter-axis dependencies consistently yields the lowest *R* ^2^ scores, underscoring its higher representational complexity and increased difficulty in generalization.

The GBM model demonstrates exceptional predictive accuracy for revolute joints and robust performance for prismatic joints, with sub-millimeter to low-millimeter error margins across datasets. These results position GBM as a highly effective, data-driven solution for inverse kinematics in modular C-arm systems. Its ability to generalize across diverse joint types makes it particularly well-suited for integration into real-time control systems and AI-assisted surgical trajectory planning frameworks, advancing the state of intelligent medical robotics.

The Predicted vs. True joint outputs on the test set are illustrated in the Supplementary Figures S1–S7 for all nine joints under the 9-DoF configuration, offering a temporal comparison across 100 representative samples. These plots provide a clear visual interpretation of each model’s fidelity in approximating the joint trajectories. The tight tracking of prediction lines to the true values in these plots reinforces the quantitative findings from MAE, RMSE, and *R* ^2^ analyses. Conversely, any observable phase shift, amplitude discrepancy, or increased oscillation between predicted and true lines in specific joints offers valuable insight into joint-specific limitations and opportunities for further model refinement.

Due to the exhaustive nature of the evaluation, and for brevity, we have included all results using ML on both forward and inverse kinematics, from 5DoF to 9DoF. These may be found in the Supplementary Material - All Results tables. To contextualize these performance levels against physically validated kinematic solutions, a direct baseline comparison with the analytical Denavit–Hartenberg (DH) formulation and its numerically validated Simscape Multibody solver from our prior work (Jaheen et al., 2025a) is presented in the following subsection.

### 4.3 Baseline comparison with analytical and numerical solvers

To establish a rigorous baseline for the ML predictions, comparisons were performed against the analytical and numerically validated solvers developed in our prior work (Jaheen et al., 2025a). In that study, the forward kinematics of the isocentric C-arm with patient table were formulated analytically using the Denavit–Hartenberg (DH) parameter method providing a closed-form expression for the end-effector transformation matrix $T_{ee}^{base}$ . The analytical results were subsequently validated numerically using a high-fidelity Simscape Multibody solver, where thousands randomly generated joint configurations were simulated to compute end-effector poses. The rotational outputs were expressed in quaternion form ( $q_{w}$ , $q_{x}$ , $q_{y}$ , $q_{z}$ ) and the translational components as ( $t_{x}$ , $t_{y}$ , $t_{z}$ ). Comparative analysis between the analytical and numerical results revealed zero rotational and translational error, confirming the deterministic accuracy of the DH-based analytical formulation and validating the Simscape solver as a reliable numerical reference.

In the present study, these validated analytical-numerical results served as ground-truth baselines for assessing the accuracy of the ML regression models developed for the 9DoF C-arm system. The quaternion data from both input and output datasets were converted to Euler angles ( $r_{x}$ , $r_{y}$ , $r_{z}$ ) to align with the pose representation used in the ML framework. As summarized in Table 6, the numerical comparison between the analytical and numerical solvers exhibited negligible orientation deviations, with both the mean absolute error (MAE) and root mean squared error (RMSE) effectively equal to zero degrees confirming complete rotational consistency between the two formulations; translational discrepancies remained sub-millimetric with RMSE values of 0.00282 mm, 0.00438 mm, and 0.00845 mm along the x, y, and z-axes respectively. While baseline errors for lower DoF modular configurations (5–8 DoF) have been comprehensively presented in earlier work (Jaheen et al., 2025a), the present validation confirms that the extended 9DoF C-arm system architecture preserves geometric fidelity and orientation precision across all degrees of freedom.

**TABLE 6:** Baseline validation between analytical and numerical solvers for the 9-DoF C-arm system.

Metrics	$r_{x}$	$r_{y}$	$r_{z}$	$t_{x}$	$t_{y}$	$t_{z}$
MAE	0	0	0	0.00026	0.00314	0.00340
RMSE	0	0	0	0.00282	0.00438	0.00845

By incorporating both analytical and numerical baselines, the study establishes a rigorous foundation for verifying the accuracy, stability, and generalizability of the proposed data-driven framework. The analytical DH-based formulation provides a deterministic and interpretable reference for kinematic relationships, while the numerical solver ensures high-fidelity validation under realistic multibody dynamics and computational perturbations. Together, these baselines confirm that the model preserves both geometric exactness and physical consistency across all degrees of freedom. Among all machine learning models evaluated in this study, the Gradient Boosting Machine (GBM) and the Deep Neural Network (DNN) integrated with Principal Component Analysis (PCA) most effectively replicated the deterministic DH-based analytical formulation, achieving near-perfect correspondence with the validated numerical solver. These models extend the applicability of the analytical baseline to complex and redundant clinical workspaces where closed-form solvers become computationally prohibitive. This dual validation underscores the numerical fidelity, physical interpretability, and scalability of the GBM- and DNN-based machine learning framework for advanced C-arm kinematic modeling.

### 4.4 Robustness analysis with perturbed inputs

Among the five evaluated regression models: Ridge Regression, K-Nearest Neighbors, Random Forest, Gradient Boosting Machine (GBM), and Deep Neural Network (DNN), the GBM consistently achieved near-zero error and a perfect coefficient of determination (*R* ^2^ ≈ 1.0) across all pose components of 9DoF C-arm system. Although this accuracy highlights its strong learning capability, it also warrants verification against possible overfitting or data-leakage artifacts. Therefore, robustness evaluation was performed exclusively for the GBM, as its ensemble-boosting architecture is particularly sensitive to distributional shifts. Assessing GBM under noisy and perturbed inputs provides the most stringent diagnostic of the framework’s numerical stability and generalization strength.

To emulate real-world uncertainties arising from encoder drift, sensor noise, and mechanical backlash, additive Gaussian perturbations were introduced into the standardized test features in Equation 6:

$$X_{noisy}=X_{test}+N\left(0,\sigma^{2}\right)$$ (6)

with σ ∈ {0.01, 0.05, 0.10} representing low, moderate, and high perturbation levels. Each perturbed dataset was scaled using the same normalization parameters derived from the training subset to avoid leakage. For each σ level, the model’s predictions were compared with ground-truth pose values using Mean Absolute Error (MAE), Root Mean Squared Error (RMSE), and Coefficient of Determination (*R* ^2^).

**FIGURE 31:**
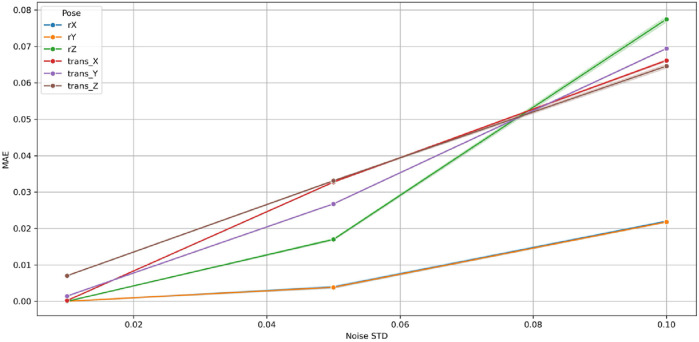
Variation of Mean Absolute Error (MAE) with Gaussian noise standard deviation (σ) for the Gradient Boosting Machine (GBM) across six pose components in the 9-DoF configuration.

**FIGURE 32:**
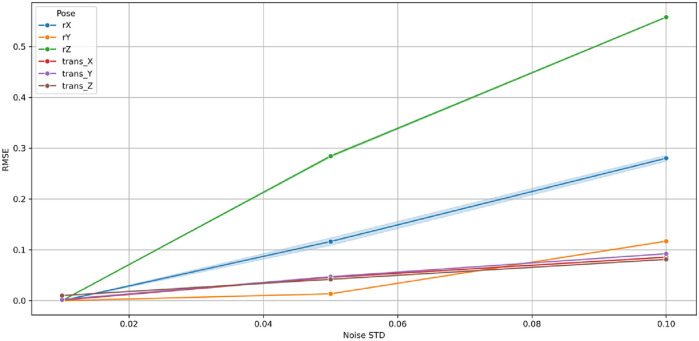
Variation of Root Mean Squared Error (RMSE) with Gaussian noise standard deviation (σ) for the GBM across six pose components in the 9-DoF configuration. The linear and bounded RMSE growth confirms predictable sensitivity to input perturbations.

**FIGURE 33:**
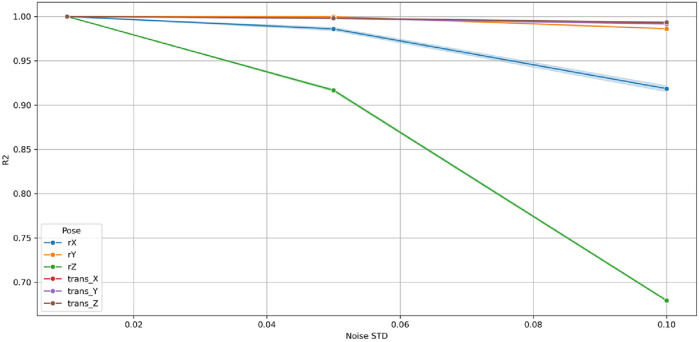
Variation of coefficient of determination (*R* ^2^) with Gaussian noise standard deviation (σ) for the GBM across six pose components in the 9-DoF configuration.

Figures 31–33 illustrate the variation of MAE, RMSE, and *R* ^2^, respectively, as a function of σ across the six pose components: three rotational ( $r_{x}$ , $r_{y}$ , $r_{z}$ ) and three translational ( $t_{x}$ , $t_{y}$ , $t_{z}$ ). At minimal perturbation (σ = 0.01), the GBM maintained near-perfect performance (MAE <0.01, RMSE <0.05, *R* ^2^ ≈ 1.0) for all rotational ( $r_{x}$ , $r_{y}$ , $r_{z}$ ) and translational ( $t_{x}$ , $t_{y}$ , $t_{z}$ ) components. As σ increased, both MAE and RMSE grew smoothly and linearly, while *R* ^2^ declined monotonically yet remained high, indicating a well-conditioned and stable response to noise. The maximum MAE remained below 0.08 and RMSE below 0.6 at σ = 0.10, corresponding to <3% performance degradation well within tolerances.

Among rotational variables, r_z_ showed the steepest sensitivity (*R* ^2^ ≈ 0.70 at σ = 0.10) owing to its multi-axis coupling with translational joints in the 9-DoF architecture. Conversely, r_x_ and r_y_ remained highly stable (*R* ^2^ > 0.90), exhibiting negligible MAE and RMSE growth. The translational components (t_x_, t_y_, t_z_) maintained *R* ^2^ > 0.98 for all σ, demonstrating high resilience of prismatic motion estimation.

Collectively, these findings confirm that the GBM is robust, numerically stable, and non-overfitted. Its bounded, linear error progression and absence of abrupt degradation establish the model’s capability to sustain accurate predictions under realistic sensor perturbations. This robustness ensures reliable C-arm positioning even with moderate measurement noise, validating the suitability of Gradient Boosting Machine for real-time intraoperative pose prediction and trajectory correction.

### 4.5 Feature importance and model interpretability

To elucidate the internal mechanics of the Gradient Boosting Machine (GBM) and to substantiate the transparency of its predictive behavior, a feature-importance analysis was performed using the Mean Decrease in Impurity (MDI) criterion. This metric quantifies the average reduction in node impurity achieved when a given feature is used for data partitioning across all decision trees in the ensemble, thus serving as an interpretable proxy for each feature’s overall contribution to model performance. Higher MDI values correspond to features that induce greater reductions in residual variance and thereby exert stronger predictive influence on the target output. The resulting importance scores were normalized and plotted on a linear scale to preserve the relative magnitudes of contribution across all joint variables.

**FIGURE 34:**
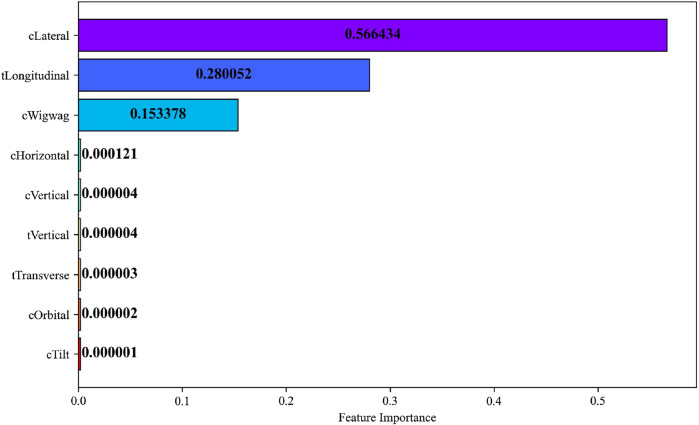
Feature importance of joint variables computed using the mean decrease in impurity (MDI) metric.

Each horizontal bar in Figure 34 represents an individual joint variable, color-coded to visually distinguish among features. Numerical annotations on the bars convey the exact MDI values, ensuring quantitative clarity and reproducibility. The visualization reveals a distinct hierarchy in feature relevance: the C-Arm Lateral, Table Longitudinal, and C-Arm Wigwag joints exhibit substantially higher MDI values 0.566, 0.280, and 0.153, respectively demonstrating their dominant influence on end-effector pose prediction. In contrast, the remaining joints possess near-zero importance values indicating negligible contributions to the model’s predictive variance.

The analysis revealed that translational joints particularly the C-Arm Lateral, Table Longitudinal, exhibited the highest MDI values underscoring their dominant role in determining spatial alignment within the surgical workspace. In contrast, joints responsible for fine-tuning adjustments displayed lower importances indicating subsidiary contributions primarily associated with local pose refinement. This hierarchy corroborates the predominance of planar and longitudinal translations in defining workspace variability and highlights the capability of the Gradient Boosting Machine to learn physically interpretable motion patterns.

By incorporating impurity-based interpretability, the proposed framework extends beyond numerical accuracy to provide transparent insights into the hierarchical structure of learned kinematic dependencies. Such interpretability not only reinforces the model’s reliability but also aligns with established variable-selection methodologies that exploit impurity-reduction indices, namely, Mean Decrease Accuracy (MDA) and Mean Decrease Gini (MDG) for feature relevance evaluation (Han et al., 2016). The integration of MDI-based importance analysis thus substantiates the robustness and explainability of ensemble learning in high-dimensional robotic regression tasks.

### 4.6 Post-prediction validation findings

To assess the physical feasibility and safety of the predicted joint configurations, a workspace validation is performed using a simulation-based collision analysis framework in Blender. This step is designed to ensure that the predicted poses not only satisfy kinematic feasibility but also reside within the robot’s collision-free operational task space under real-world constraints. In this process, the predicted joint configurations obtained from each machine learning model are applied to the C-arm’s 3D kinematic structure modeled in Blender using the *Armature* and *Constraint* system. A Python script executed within the Blender environment systematically samples these joint configurations and forwards them to the robot model. The end-effector poses are then computed and analyzed for collisions with both the robot’s own structure and environmental boundaries, leveraging Blender’s built-in rigid body physics engine for precise detection.

Table 7 reports the percentage of predicted configurations that successfully fall within the collision-free workspace, across various DoF reductions. For the full 9DoF configuration, 100% workspace validity is achieved across all models, confirming the physical plausibility of learned solutions. As dimensionality is reduced, slight degradation in workspace coverage is observed for a few models most notably at 5DoF, where linear regression and tree-based models such as RF and GBM drop to 99.48% and 99.82% respectively. Conversely, deep neural networks, particularly the DNN with PCA, maintain 100% validity even in underactuated conditions.

**TABLE 7:** Percentage of collision-free poses across machine learning models for 5DoF to 9DoF configurations.

Configuration	Linear regression	Gradient boosting machine (GBM)	K-Nearest neighbor (KNN)	Random forest (RF)	Deep neural network (DNN-128-64-32)	Deep neural network (DNN-256-128-64)	Deep neural network (DNN-256-128-64) with PCA
9DoF	100%	100%	100%	100%	100%	100%	100%
8DoF	100%	100%	100%	100%	100%	100%	99.99%
7DoF	99.99%	99.99%	100%	100%	100%	100%	100%
6DoF	99.99%	99.99%	100%	99.99%	100%	100%	100%
5DoF	99.41%	99.48%	99.82%	99.48%	100%	99.82%	100%

Following the validation of predictive accuracy and pose consistency, a dedicated analysis was conducted to evaluate the computational efficiency of the two most performant models Gradient Boosting Machine (GBM) and Deep Neural Network (DNN) with PCA. This assessment quantifies both training and robustness-testing runtimes for each pose variable under identical hardware and software conditions, providing an empirical measure of algorithmic scalability and real-time feasibility. The corresponding results and comparative interpretation are presented in the following subsection.

### 4.7 Computational cost and runtime analysis

To evaluate computational efficiency and assess real-time feasibility, a comprehensive runtime analysis was conducted for the two most accurate models Gradient Boosting Machine (GBM) and Deep Neural Network (DNN) with PCA applied to the 9-DoF C-arm with operating table configuration. This configuration was deliberately selected because it represents the maximum kinematic complexity within the modular system, combining a 6-DoF enhanced C-arm with a 3-DoF surgical table. As the highest-order model, it encapsulates the complete set of translational and rotational couplings, redundancy, and nonlinear dependencies inherent to the integrated architecture. Evaluating computational cost under this scenario therefore provides a conservative upper bound on runtime and scalability, ensuring that all lower-DoF configurations would entail proportionally lower computational requirements.

The computing environment and framework specifications are provided in Section 3.F (System Configuration and Training Methodology). Each pose component three rotational (r_x_, r_y_, r_z_) and three translational (t_x_, t_y_, t_z_) was trained and validated independently under identical hardware and software conditions to ensure methodological uniformity.

Table 8 summarizes the total time, encompassing the training, validation, and robustness evaluation phases for both Gradient Boosting Machine and Deep Neural Network with PCA. Although the two architectures achieved similar predictive accuracy, their computational characteristics diverged significantly. The Gradient Boosting Machine model exhibited longer training durations due to its sequential additive boosting process, wherein each weak learner (decision tree) is iteratively fitted to the residual errors of preceding learners. Conversely, the Deep Neural Network with PCA leveraged vectorized GPU-accelerated tensor operations for parallelized backpropagation, achieving markedly faster convergence despite having over 100,000 trainable parameters.

**TABLE 8:** Empirical runtime analysis of GBM and DNN models under identical computational settings for the 9-DoF configuration.

Pose	Runtime of deep neural network with PCA (min: sec)	Runtime of gradient boosting machine (min: sec)
r_x_	02:22	09:13
r_y_	01:44	07:28
r_z_	03:00	09:55
t_x_	01:12	12:47
t_y_	01:12	12:37
t_z_	01:07	12:24

The cumulative runtime indicates that the DNN with PCA achieved approximately a six-fold speed-up relative to GBM under identical conditions. This improvement arises from the intrinsic parallelizability of neural computations and the exploitation of GPU-based acceleration, in contrast to the inherently serial tree construction of boosting ensembles. Despite the larger parameter count, the DNN achieved faster optimization due to efficient gradient-based learning and adaptive learning-rate scheduling.

Furthermore, inference latency for both architectures remained below 1 millisecond per sample, which is orders of magnitude faster than the mechanical actuation latency of contemporary robotic C-arm systems (typically 10–50 millisecond). These findings confirm that both models satisfy the real-time operational constraints required for intraoperative imaging and trajectory control. While GBM provides enhanced interpretability and robustness to outliers, the DNN offers a superior balance of accuracy, scalability, and computational efficiency, rendering it the most suitable architecture for predictive motion control and real-time pose estimation in modular C-arm systems.

Hence, the reported results for the 9-DoF configuration represent the upper computational bound for all modular architectures considered in this study, ensuring that the proposed framework remains computationally feasible across the full range of system complexities.

### 4.8 Clinical validation and path toward physical validation

The proposed machine-learning framework is inherently designed for unified integration into clinical C-arm fluoroscopy workflows and for future deployment on physical hardware platforms. All simulations were executed under strict mechanical range-of-motion constraints, reflecting the translational and angular limits (in meters and degrees) of commercially available C-arm and operating table systems. These parameters were derived from the manufacturer specifications of GE Healthcare and STERIS, ensuring that every simulated configuration remains mechanically feasible and directly reproducible on real devices.

While simulation-based validation confirmed that all predicted joint configurations remained within collision-free and kinematically feasible regions, the transition from virtual evaluation to clinical implementation necessitates careful consideration of hardware-specific dynamics and calibration fidelity. In practice, positioning inaccuracies in C-arm fluoroscopy arise primarily from device-level factors rather than anatomical deformation. These include encoder quantization, mechanical backlash, hysteresis, and constraints in smooth control of velocity, acceleration, and jerk, which can introduce minor positional drift of the imaging gantry during prolonged procedures or repeated articulations. Accordingly, the simulation environments in this study were explicitly constrained by manufacturer-defined motion limits for both the GE OEC 3D C-arm and the STERIS CMAX™ X-ray operating table. By enforcing these bounds, the generated datasets reflect mechanically realizable trajectories that can be executed by current clinical systems without violating operational constraints.

Modern C-arm fluoroscopes already incorporate quasi-automatic image-distortion correction and continuous geometric calibration routines that substantially mitigate hardware-induced errors. Residual drift, when present, typically stems from long-term mechanical wear, gravitational sag and so on. Contemporary interventional protocols address these residual errors through virtual-fluoroscopy systems that employ 2D/3D CT X-ray registration overlays. In such frameworks, preoperative CT volumes are aligned with intraoperative X-ray images by synthesizing Digitally Reconstructed Radiographs (DRRs) and iteratively optimizing projection geometry to minimize misalignment. This process provides an accurate, real-time estimation of the C-arm’s 3D pose, thereby compensating for both calibration offsets and mechanical deviations.

The present study directly builds upon these clinically validated practices. In prior investigations by Jaheen et al. (2025a), Jaheen et al. (2025b), DRR-based registration pipelines were applied for validating datasets in multiple clinical projections to assess the geometric fidelity of simulated C-arm poses. The DRR-based validation confirmed sub-millimeter alignment between reconstructed and acquired fluoroscopic projections, thereby demonstrating that the simulated kinematics faithfully reproduce real device behavior. The current machine-learning framework extends this foundation by learning kinematic mappings exclusively from datasets generated within these experimentally validated motion envelopes, ensuring both physical realism and clinical consistency. The integration of DRR-based registration within the proposed workflow is further supported by contemporary advancements in 2D/3D registration algorithms (Miao et al., 2018; Liao et al., 2019; Markelj et al., 2012), which collectively reinforce the methodological continuity between simulation and clinical imaging systems.

Although direct hardware experimentation falls outside the present study’s scope, the proposed models are readily compatible with existing C-arm calibration and tracking infrastructures. The predicted joint configurations can serve as data-driven pose priors that initialize or accelerate registration-based tracking pipelines, improving computational efficiency during intraoperative alignment. Moreover, once trained on empirical device data, the framework could function as a real-time predictive controller, continuously refining pose trajectories to ensure smooth, collision-free motion under mechanical and safety constraints.

A future hardware-in-the-loop validation environment is envisioned, wherein the predicted trajectories will be executed on a physical C-arm system and compared against encoder feedback and optically tracked end-effector poses. Such experiments will quantitatively assess the framework’s robustness to encoder noise, mechanical backlash, and actuation nonlinearity. In this regard, the proposed methodology not only bridges the gap between simulation-driven modeling and clinical translation but also establishes a scalable foundation for intelligent C-arm systems capable of real-time pose prediction, adaptive trajectory planning, and drift compensation in image-guided minimally invasive surgery. The outlined validation pathway provides a rigorous and technically feasible roadmap to transition from simulation-based development toward hardware-grounded, clinically viable implementation.

## 5 Future work

Future work will focus on embedding the proposed machine-learning framework within a Robot Operating System (ROS 2) environment to enable real-time communication, control, and interoperability with physical C-arm and surgical table hardware. ROS integration will support hardware-in-the-loop (HIL) and closed-loop trajectory execution linking the learning model directly to encoder feedback, optical tracking, and navigation sensors. This will allow adaptive compensation for mechanical drift, patient motion, and system latency during intraoperative imaging. Building on the supervised models developed in this study, a reinforcement learning (RL) module will be implemented to autonomously refine motion policies under anatomical and workspace constraints. By learning through interaction, the RL agent will enable adaptive, collision-free trajectory planning in hybrid operating rooms. To strengthen clinical interpretability, Explainable AI (XAI) methods such as SHAP-based feature attribution and saliency-guided reasoning will be integrated to visualize how each joint or table motion contributes to pose prediction. This will promote transparency and trust in data-driven decision-making. Further research will explore multi-agent robotic coordination through ROS-enabled communication among imaging devices, navigation systems, and robotic manipulators supporting synchronized positioning and tool guidance. Ultimately, clinical translation will focus on patient-specific validation through integration with preoperative imaging, 2D/3D registration, and intraoperative navigation pipelines, advancing toward a ROS-integrated, adaptive, and explainable surgical imaging ecosystem ready for deployment in next-generation operating rooms.

## 6 Conclusion

This paper presents the first comprehensive machine learning framework for solving both forward and inverse kinematics in a modular 9-DoF C-arm robotic system, encompassing translational and rotational degrees of freedom across both the imaging arm and the operating table. By leveraging a diverse range of regression models including linear baselines, ensemble methods, and deep neural networks with and without dimensionality reduction; the framework learns highly accurate mappings between joint configurations and 6D end-effector poses.

In forward kinematics, ensemble-based models and PCA-enhanced deep neural networks demonstrate superior generalization capabilities, producing highly accurate predictions for both translational and rotational components. The GBM and PCA-augmented DNN models consistently outperform other approaches in key evaluation metrics, achieving near-perfect alignment with ground truth data. These results establish their effectiveness for precision pose estimation within clinical imaging systems, where both spatial accuracy and predictive reliability are critical.

For inverse kinematics, GBM achieves exceptional prediction fidelity, particularly in rotational joint estimation, with zero-error performance alongside a consistently high level of variance explanation. In prismatic joints, the model maintains low error magnitudes and high *R* ^2^ values, effectively capturing complex pose-to-joint mappings. The feasibility of these predictions is further validated through numerical Blender-based workspace analysis, confirming that the joint outputs remain within the system’s collision-free task space and are executable under real-world constraints.

The findings highlight the potential of AI-driven kinematics modeling to replace traditional numerical solvers, reduce computational latency, and enable real-time control in high-precision medical robotics. The integration of data-driven methods with physical validation enhances the reliability of autonomous trajectory planning, especially in safety-critical surgical environments.

## Data Availability

The datasets presented in this study can be found in online repositories. The names of the repository/repositories and accession number(s) can be found in the article/Supplementary Material.
