# Supplementary material for: Modelling C-arm fluoroscopy and operating table kinematics via machine learning
Source: Front Robot AI. 2026 Feb 5;12:1691576. doi: 10.3389/frobt.2025.1691576 (PMC12917506; doi:10.3389/frobt.2025.1691576)
Supplement: Supplementary file 1 [file Supplementaryfile1.docx]

Supplementary Material - Figs. S1-S7

| 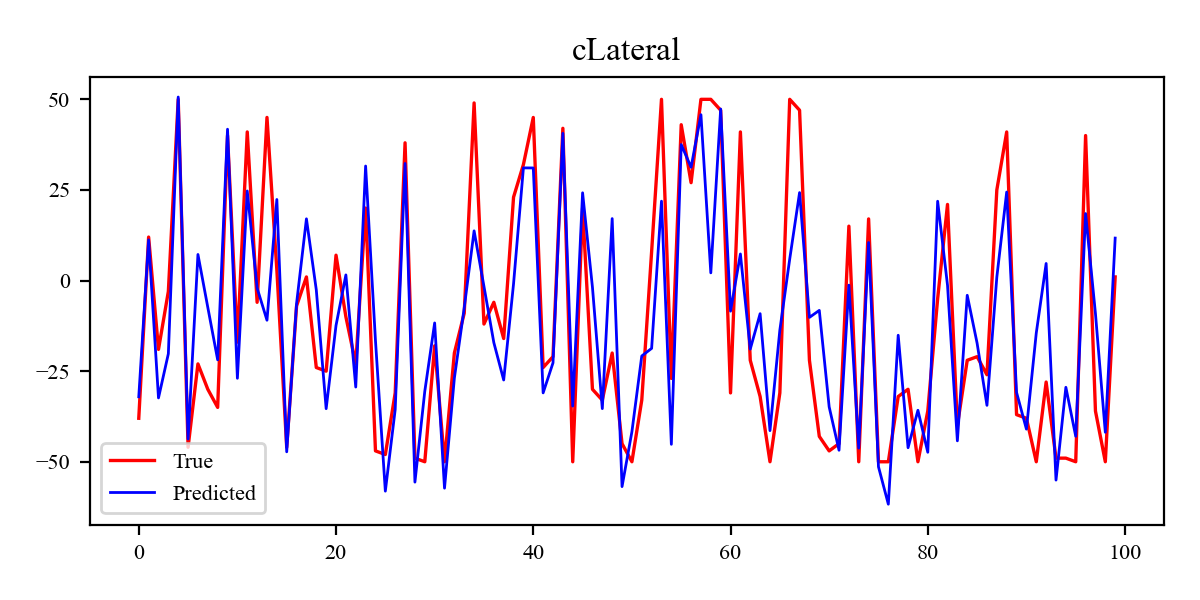 | 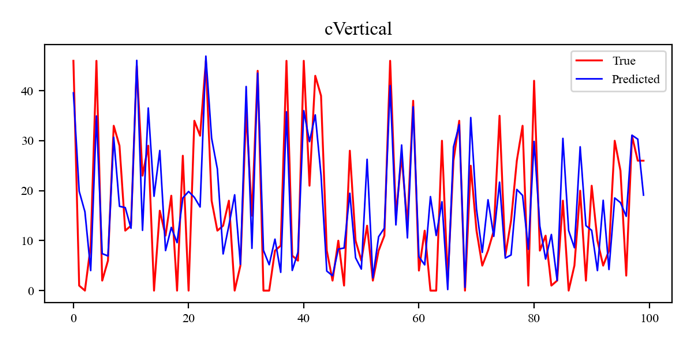 |
| --- | --- |
| 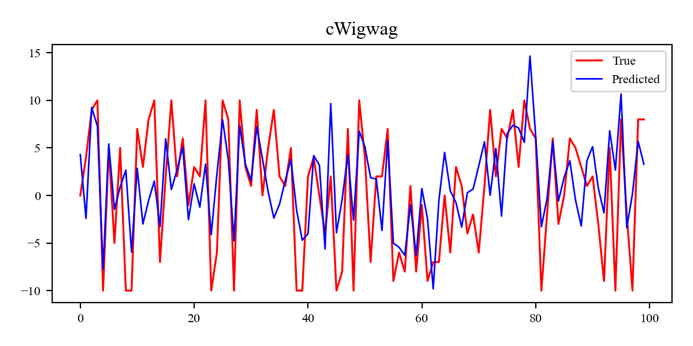 | 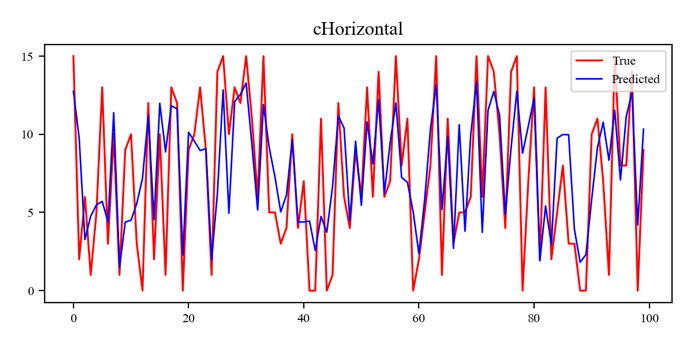 |
| 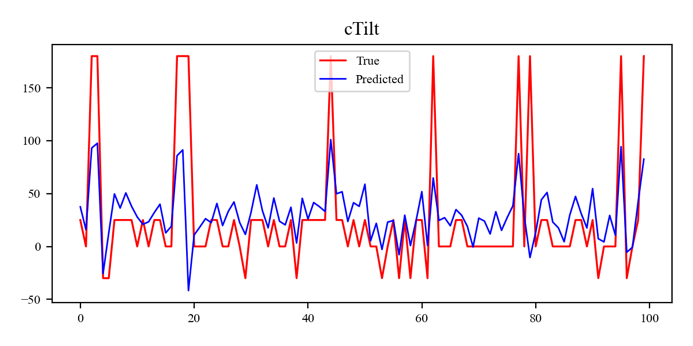 | 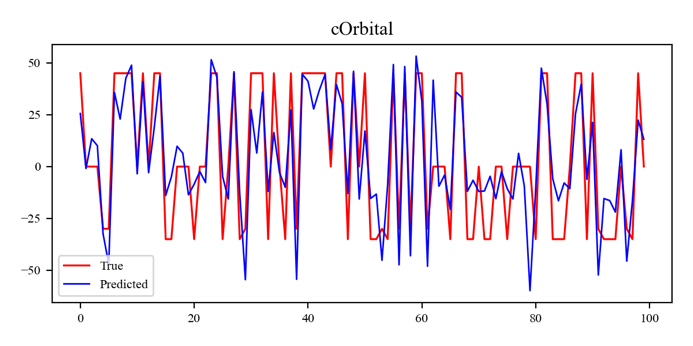 |
| 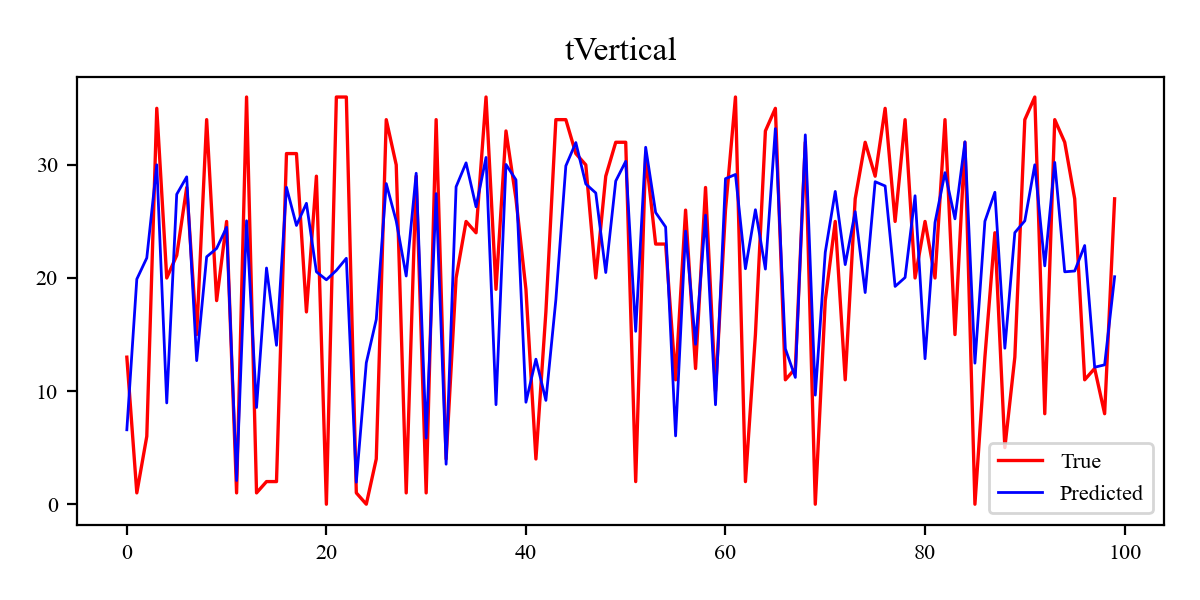 | 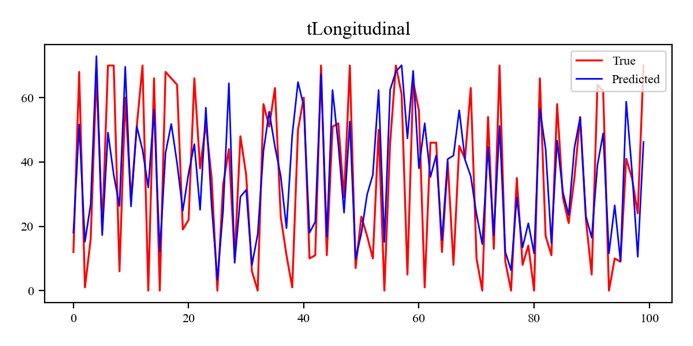 |
| 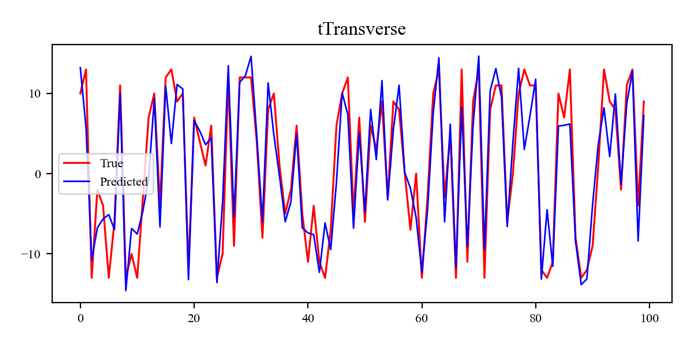 |  |

Figure S1: Prediction on the test set using Ridge Regression.

| 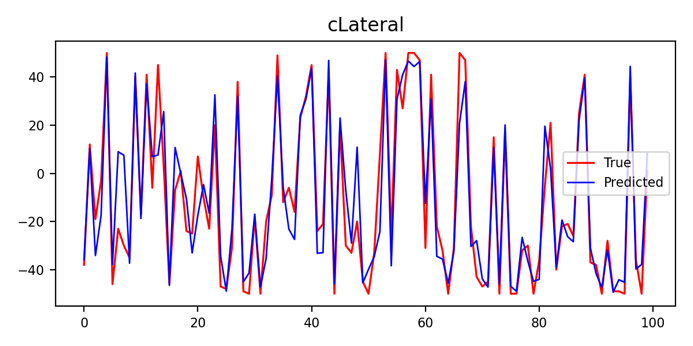 | 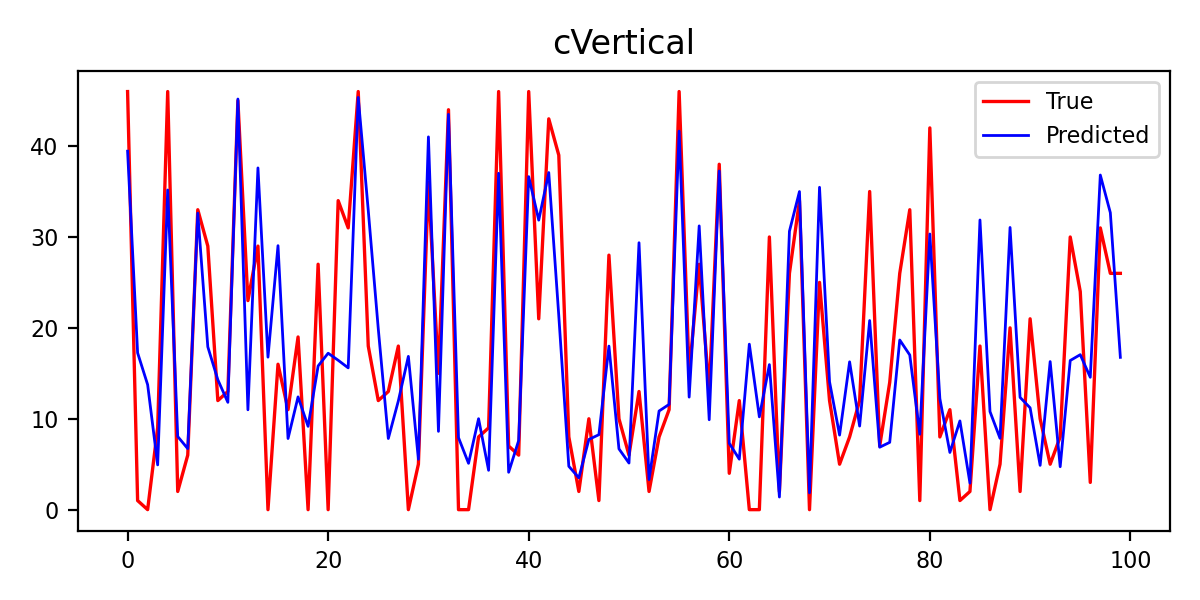 |
| --- | --- |
| 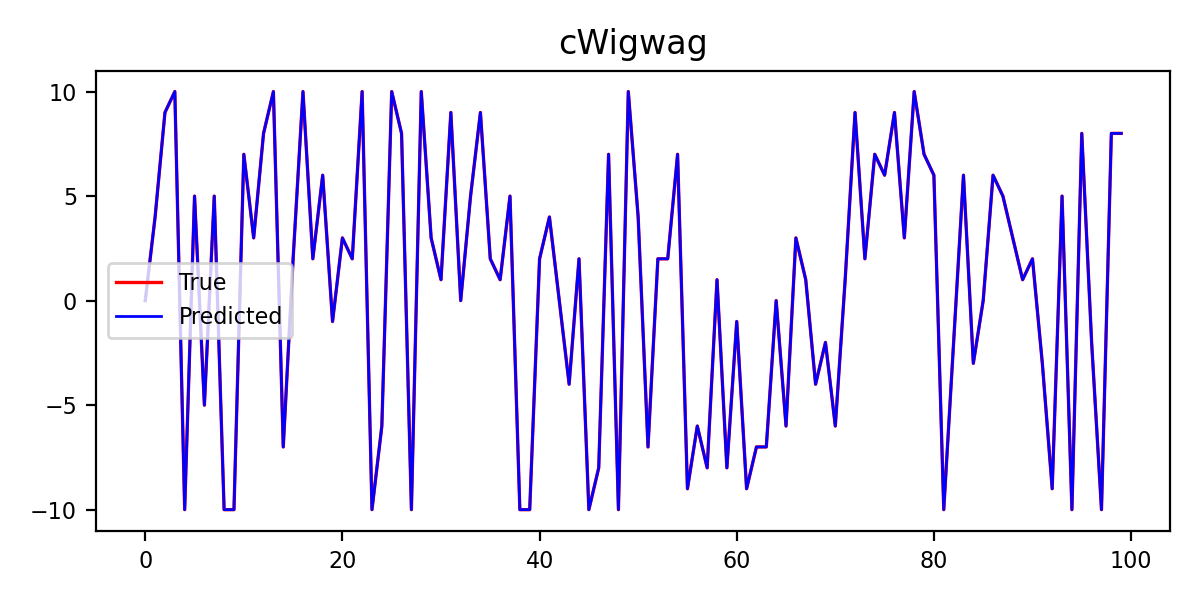 | 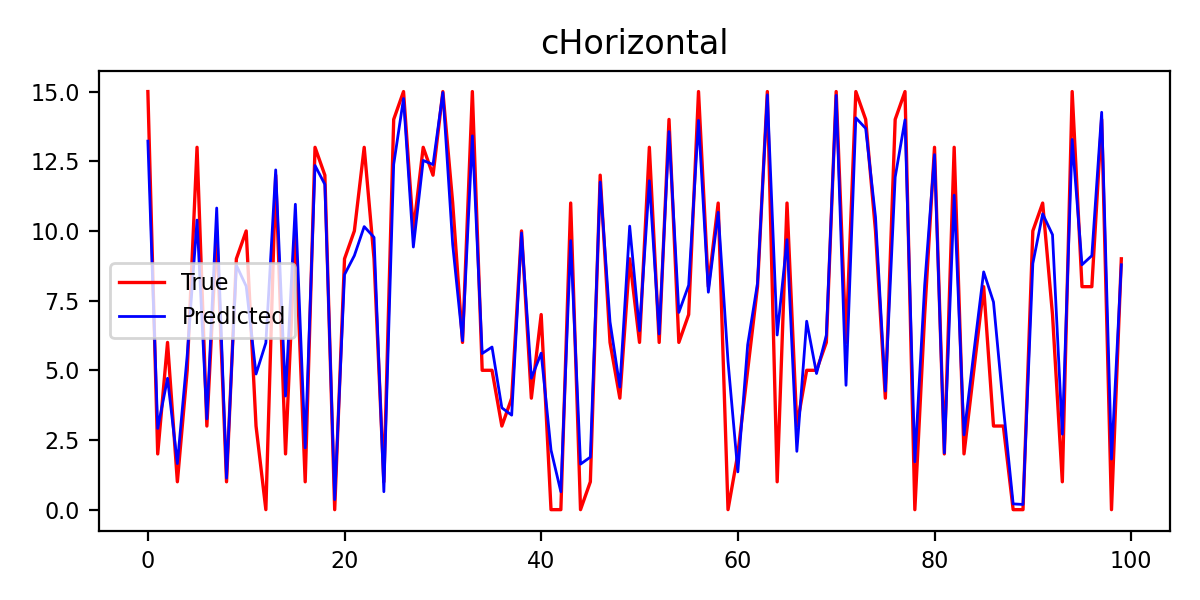 |
| 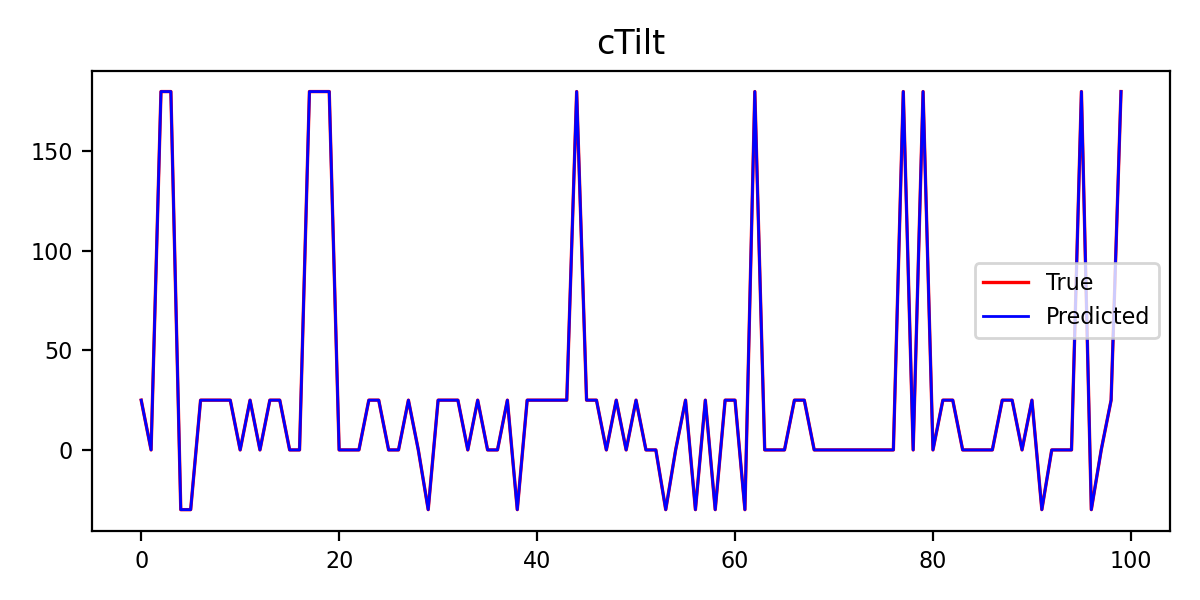 | 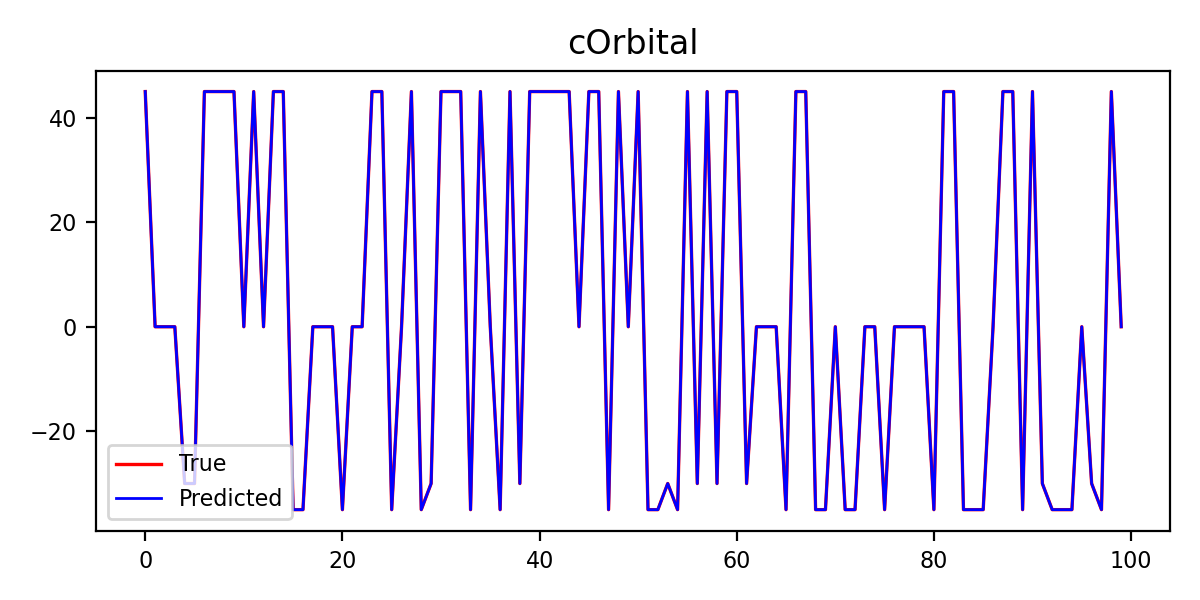 |
| 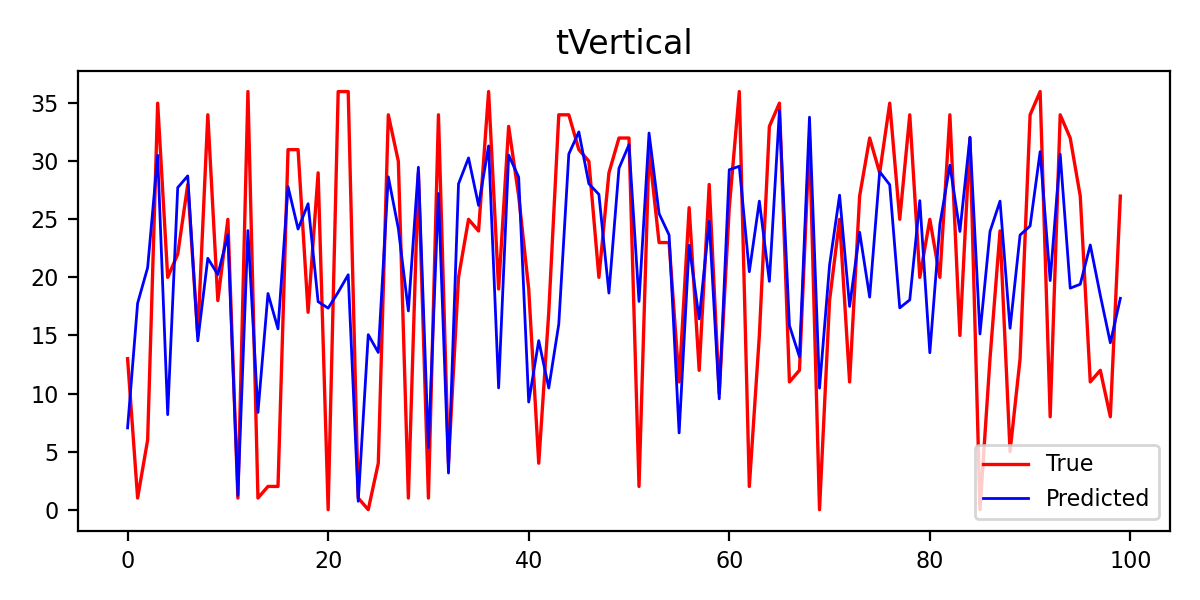 | 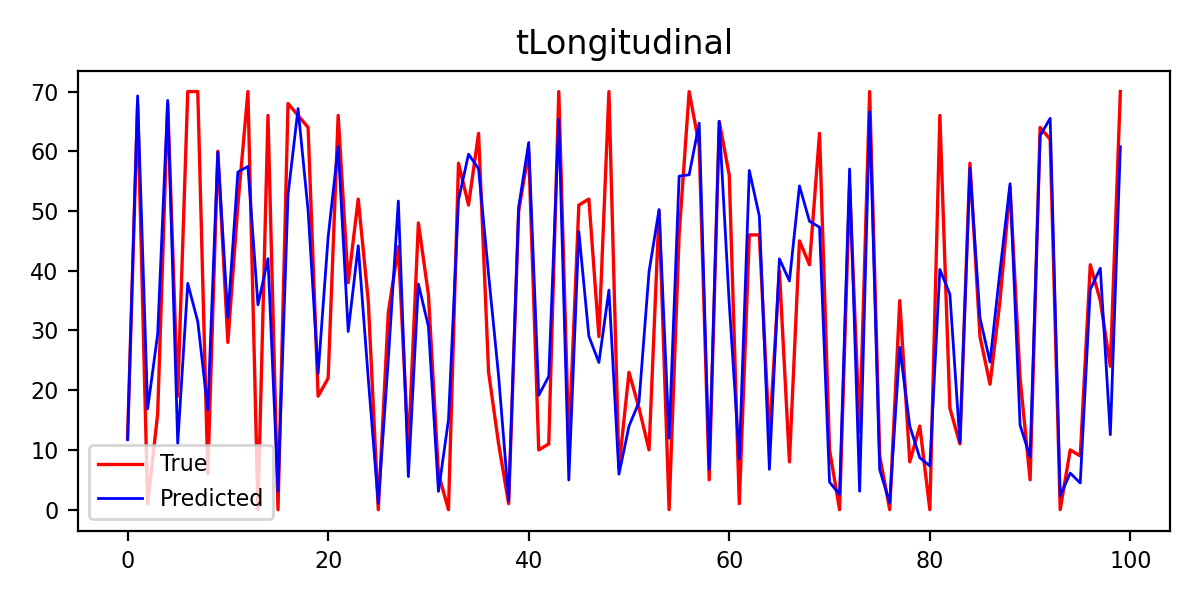 |
| 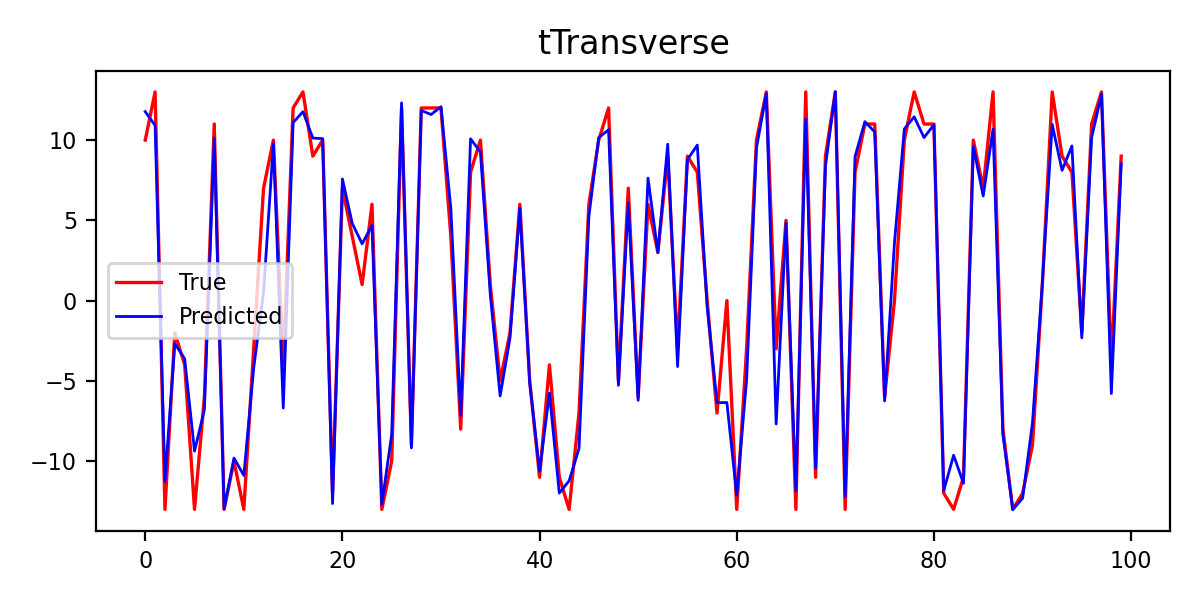 |  |

**Figure S2:** Prediction on the test set using Gradient Boosting Machine.

| 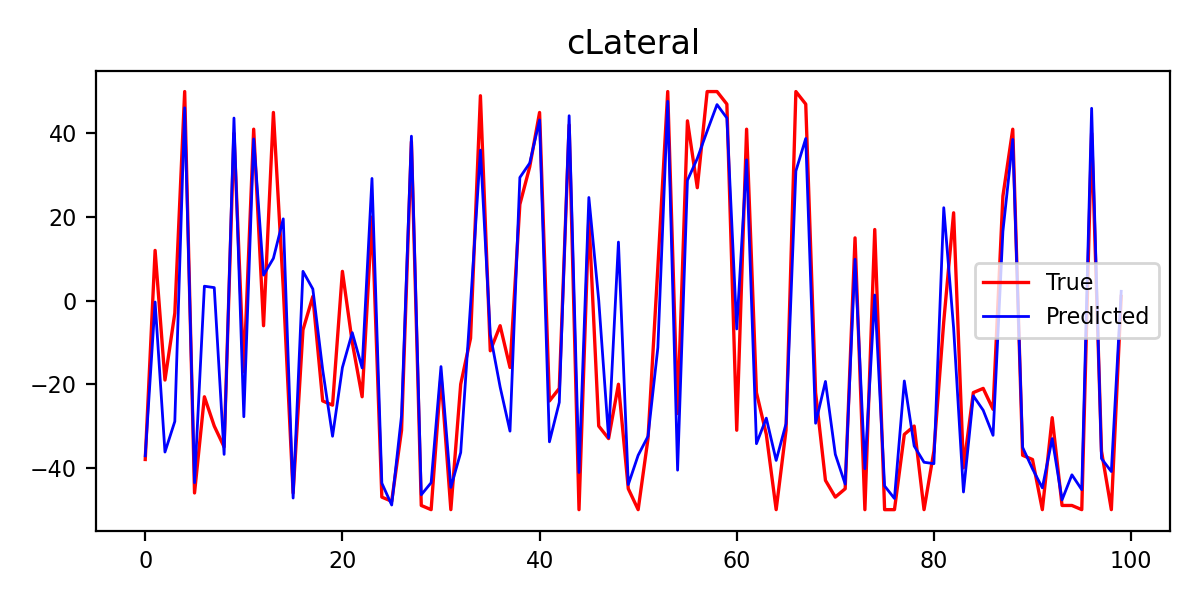 | 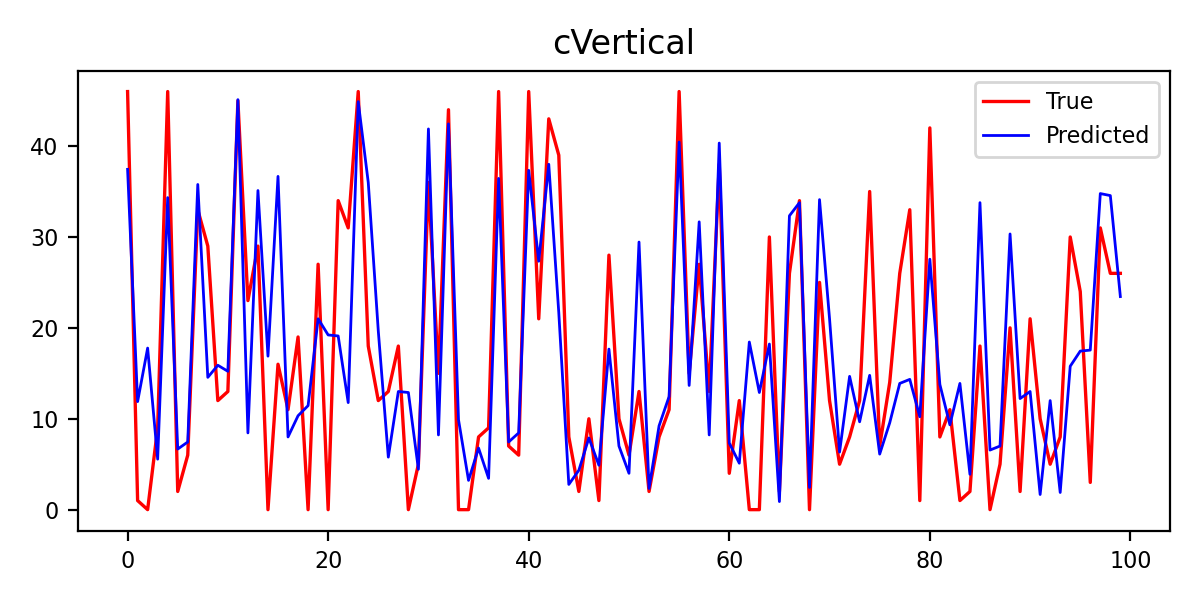 |
| --- | --- |
| 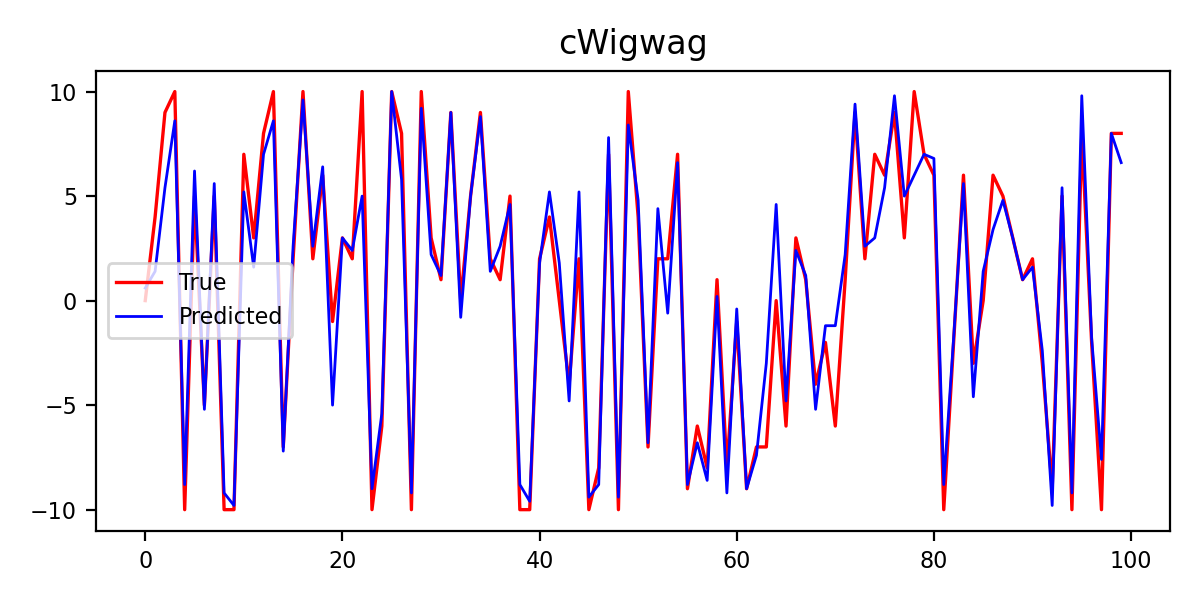 | 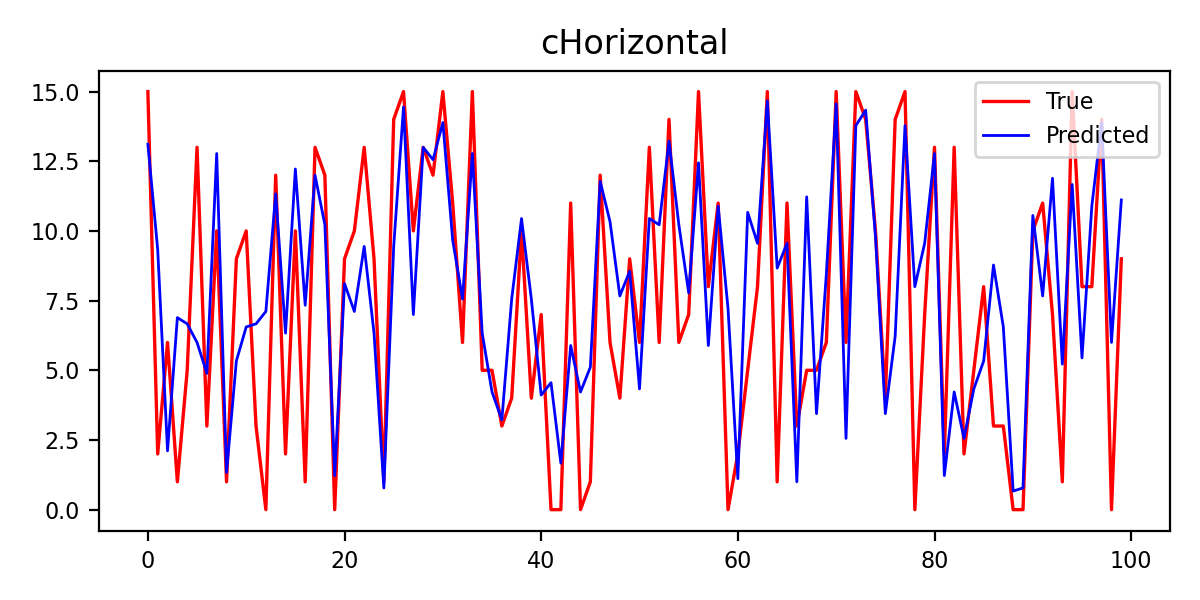 |
| 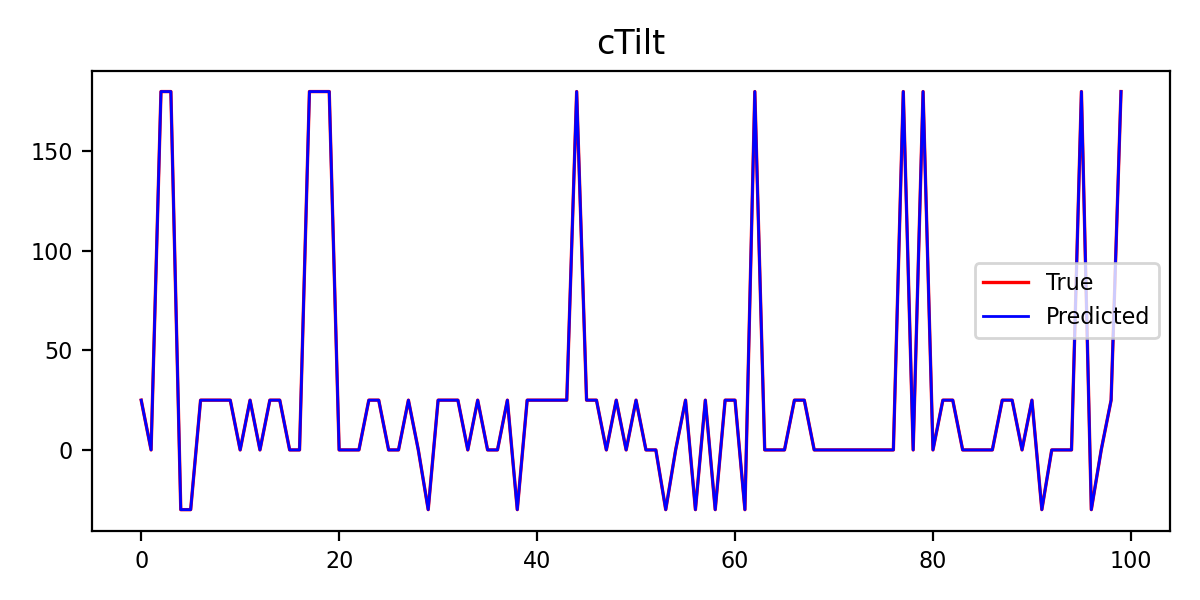 | 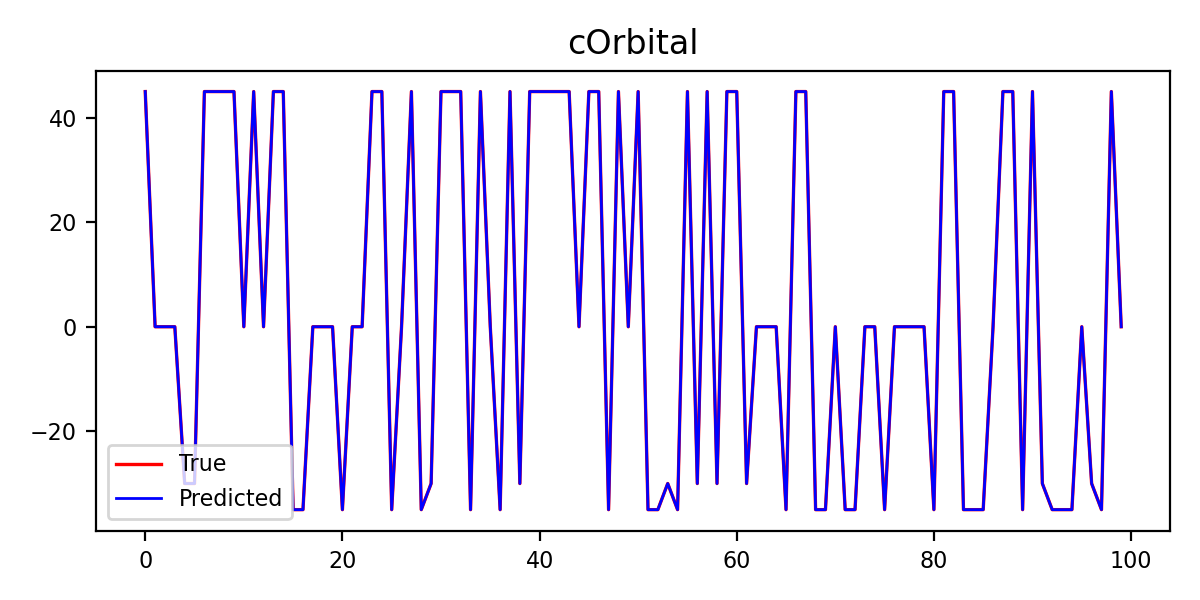 |
| 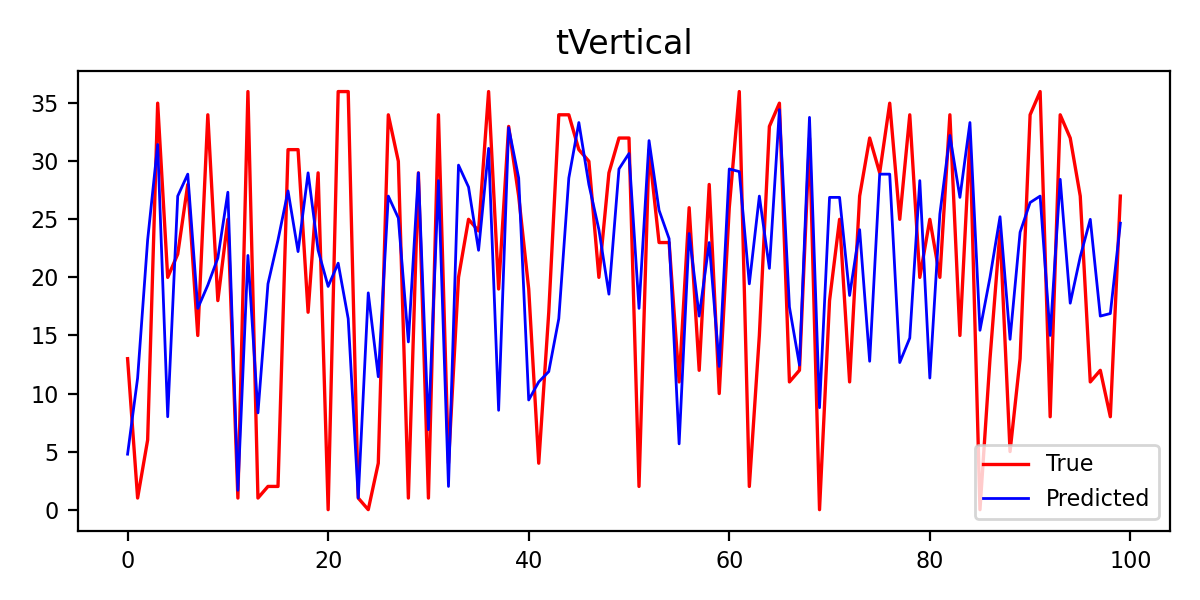 | 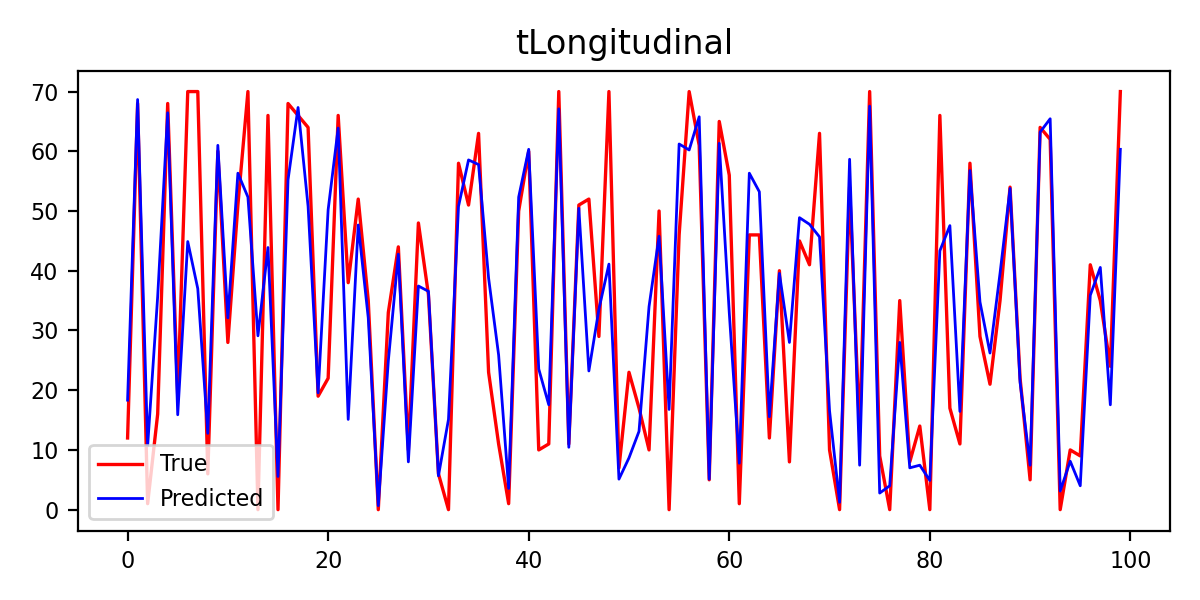 |
| 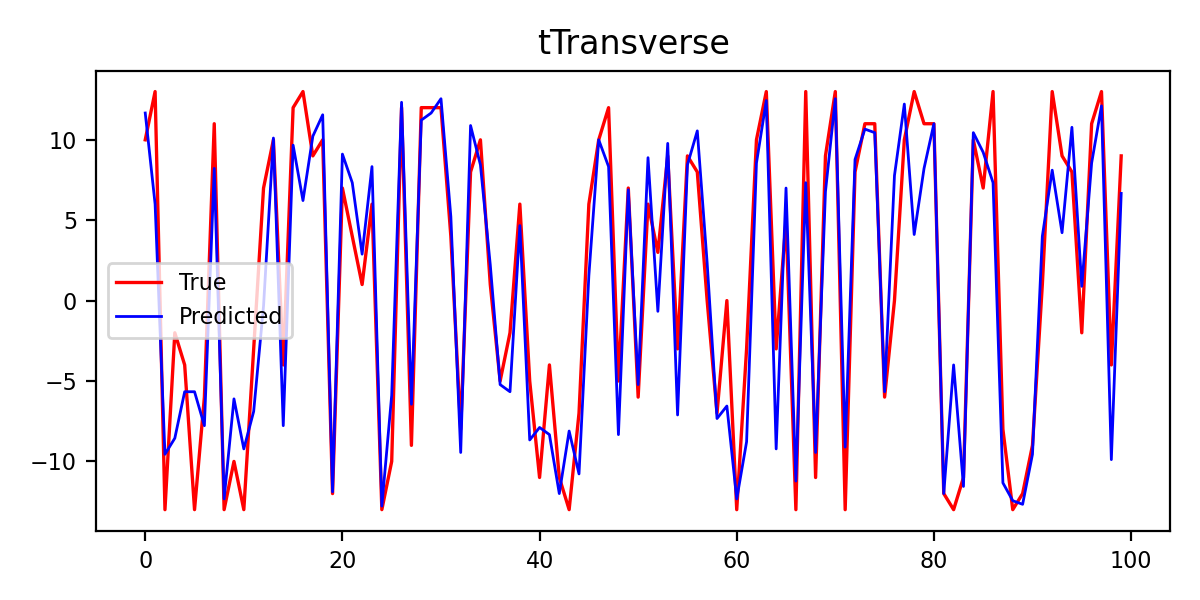 |  |

**Figure S3:** Prediction on the test set using K-Nearest Neighbors.

| 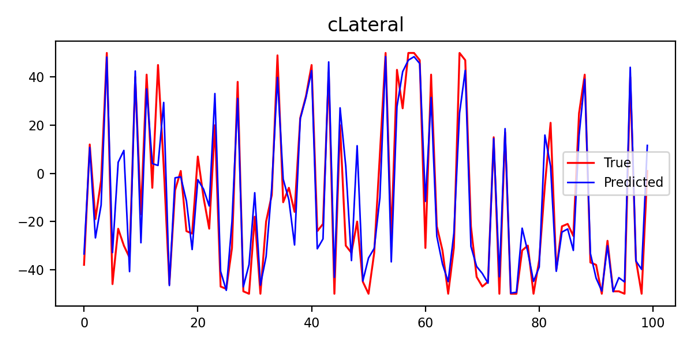 | 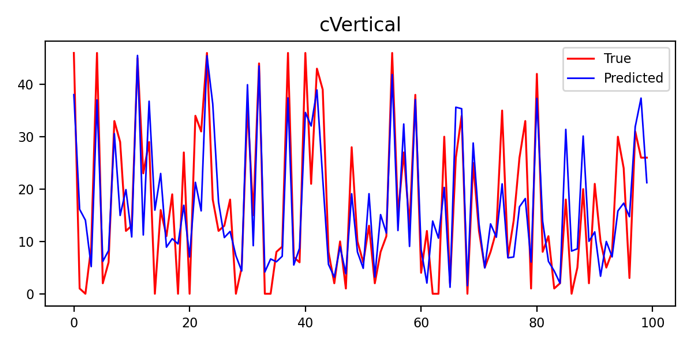 |
| --- | --- |
| 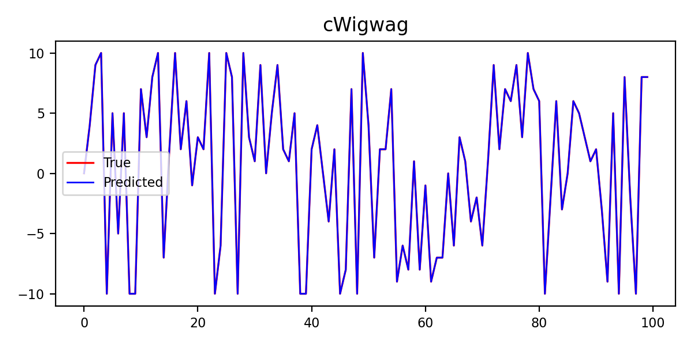 | 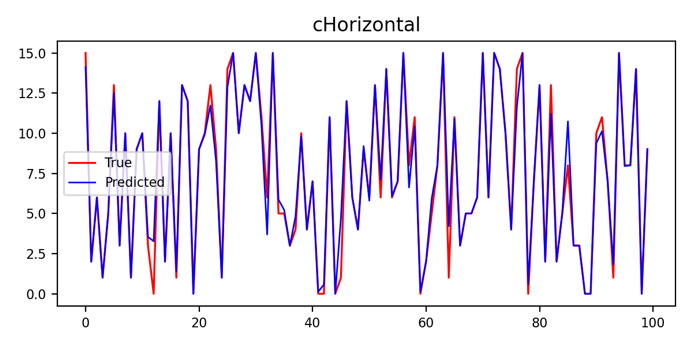 |
| 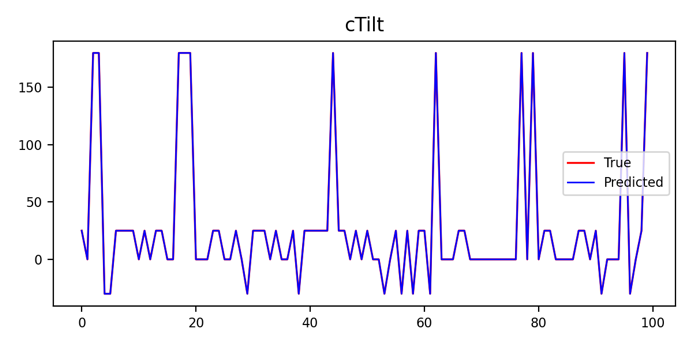 | 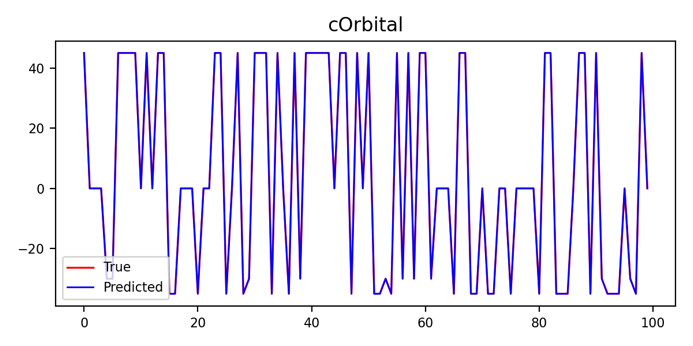 |
| 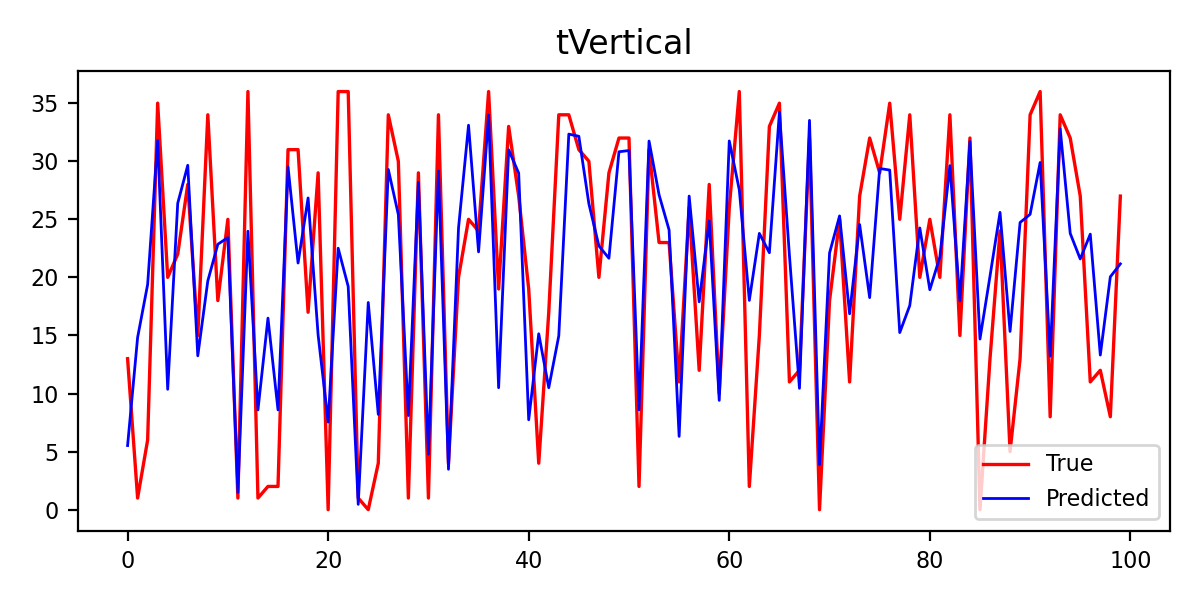 | 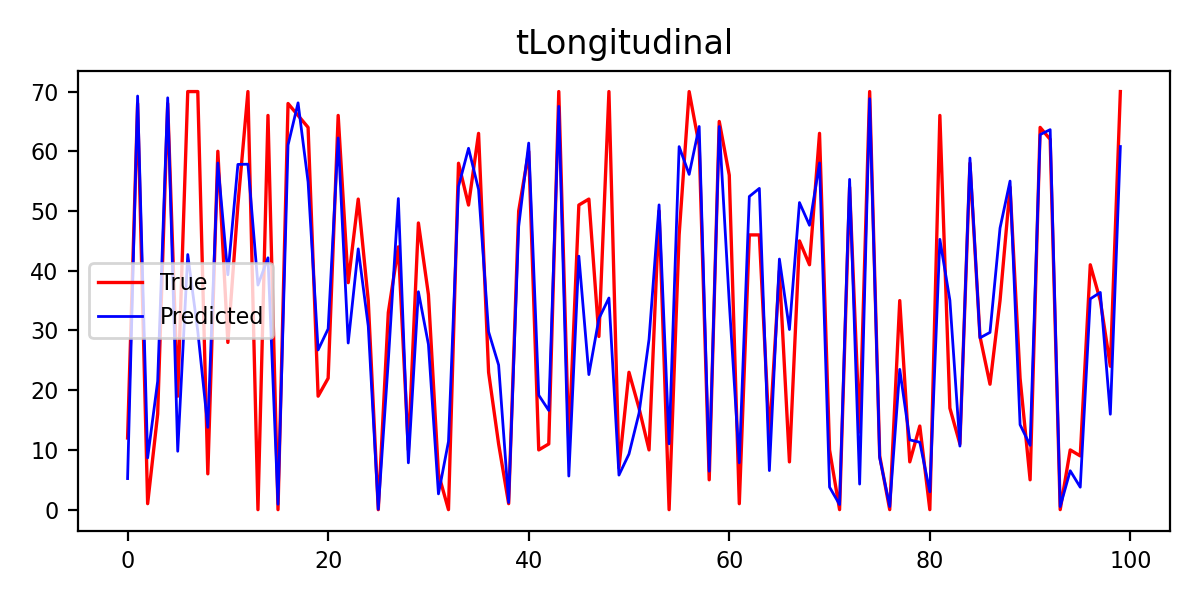 |
| 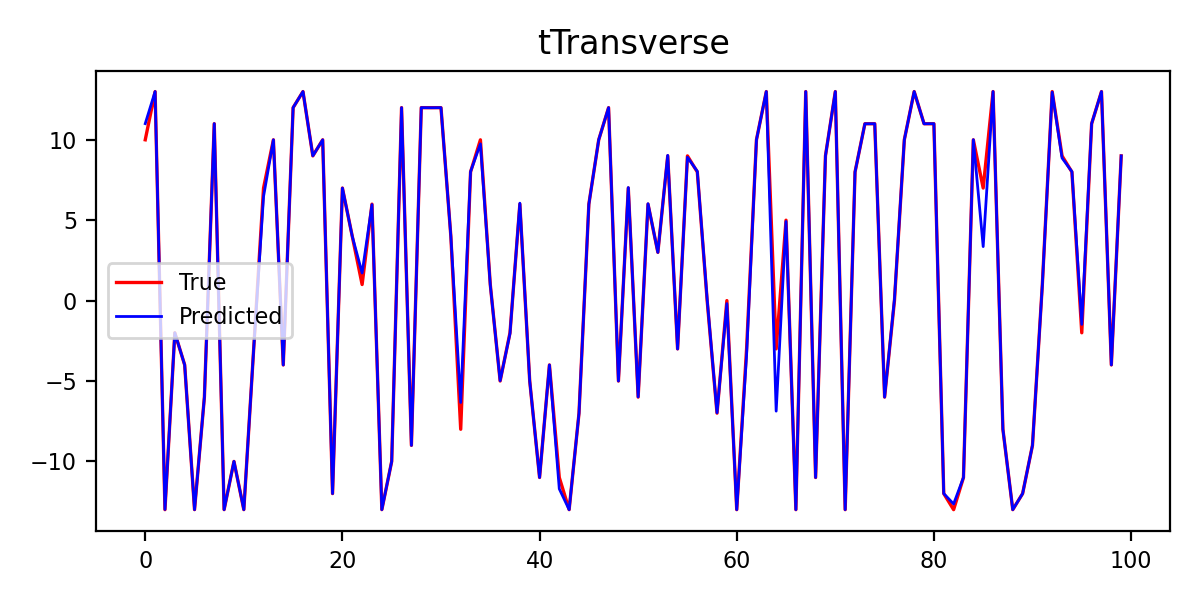 |  |

**Figure S4:** Prediction on the test set using Random Forest.

| 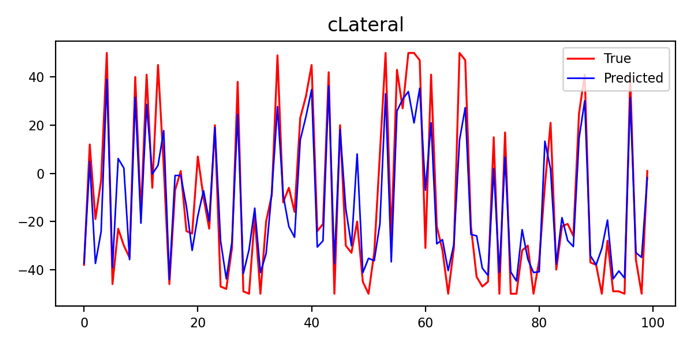 | 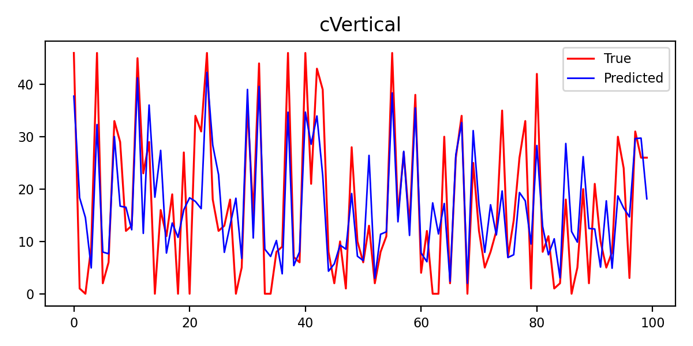 |
| --- | --- |
| 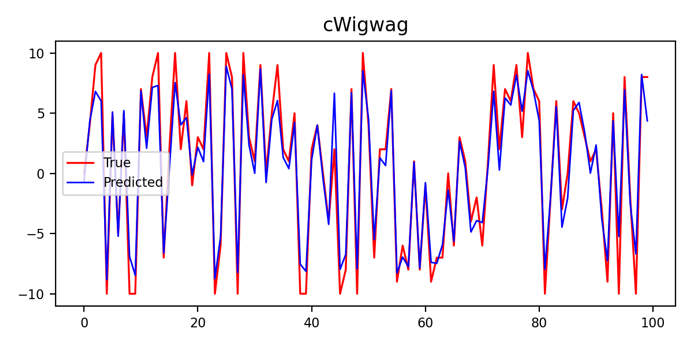 | 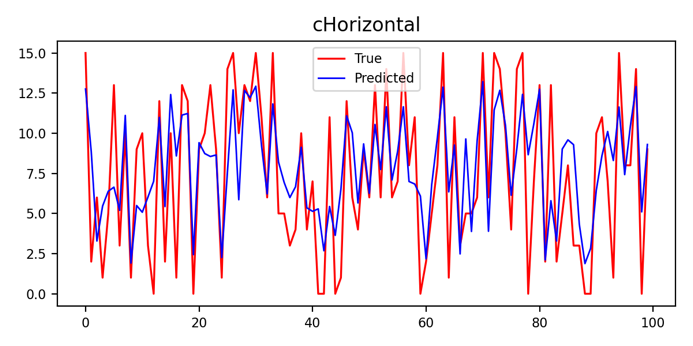 |
| 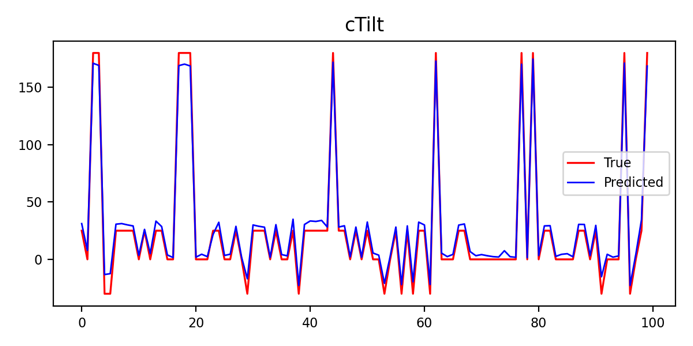 | 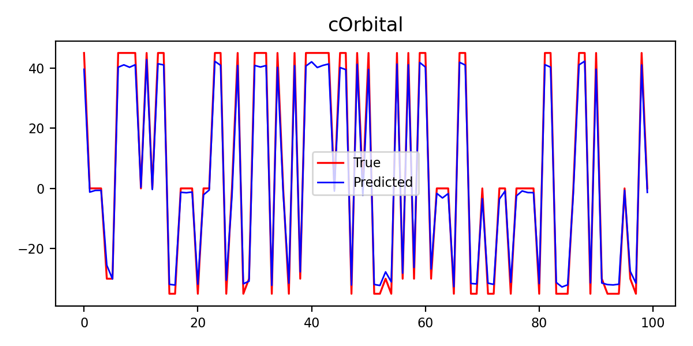 |
| 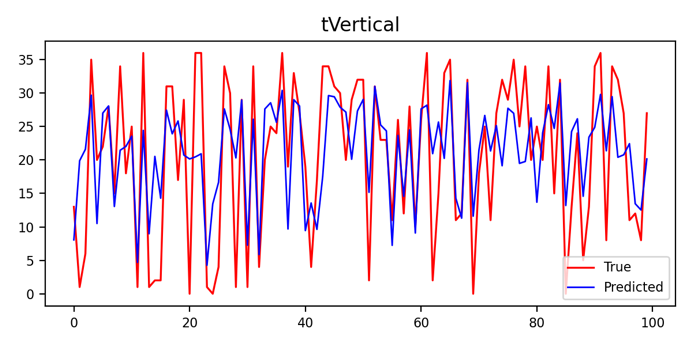 | 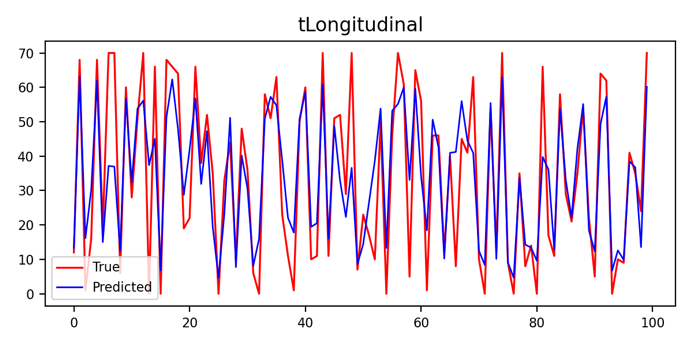 |
| 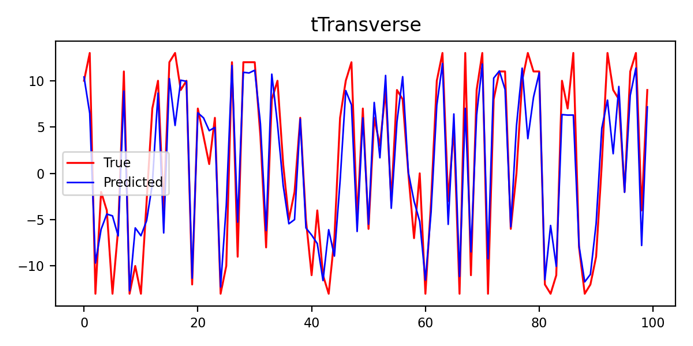 |  |

**Figure S5:** Prediction on the test set using Deep Neural Network (128–64-32).

| 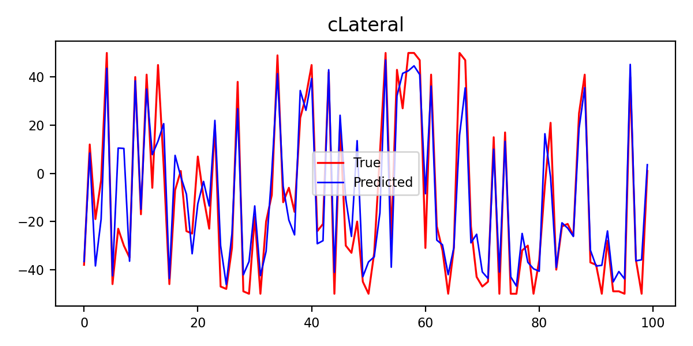 | 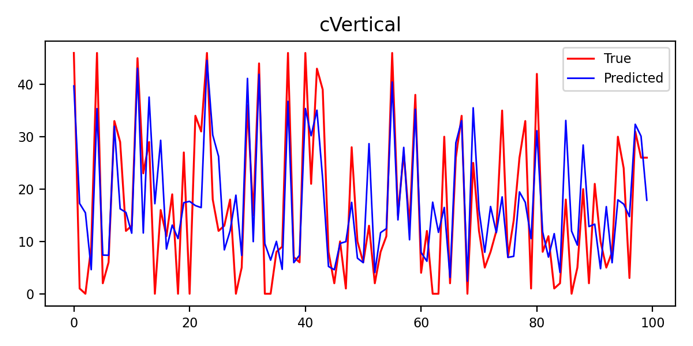 |
| --- | --- |
| 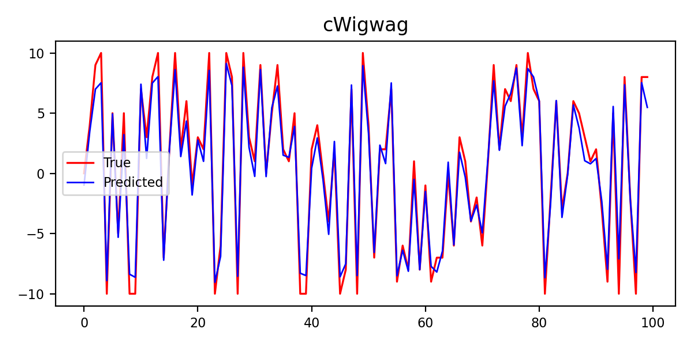 | 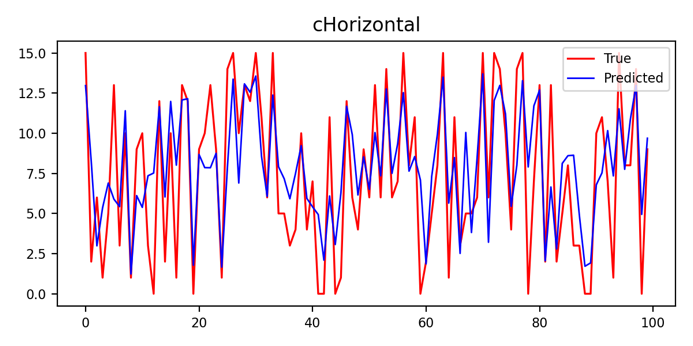 |
| 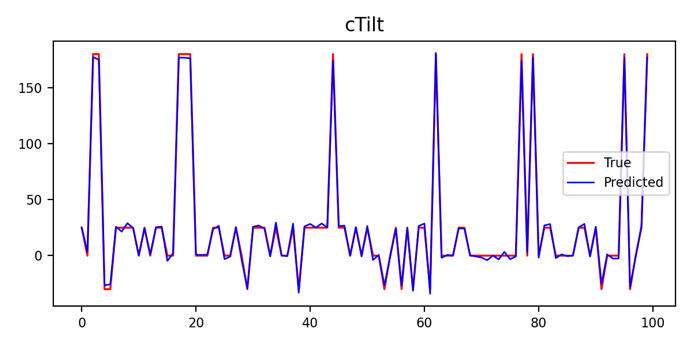 | 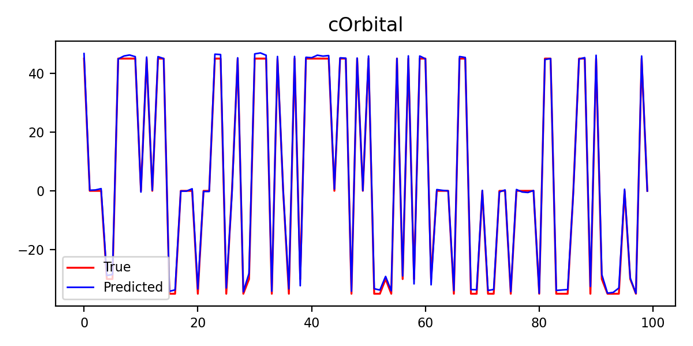 |
| 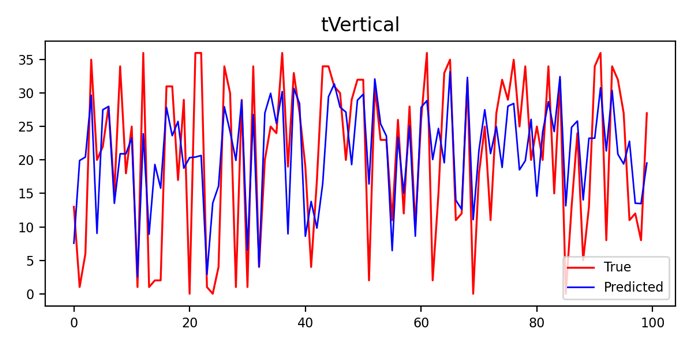 | 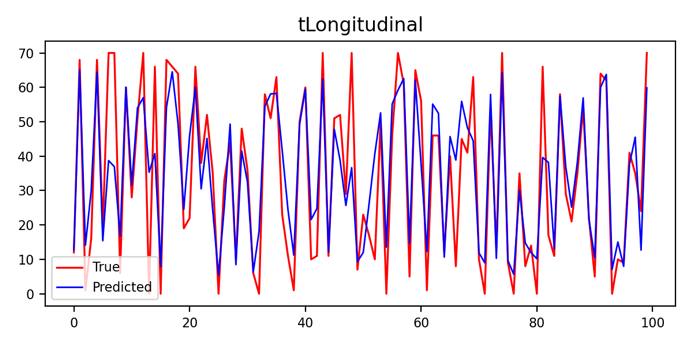 |
| 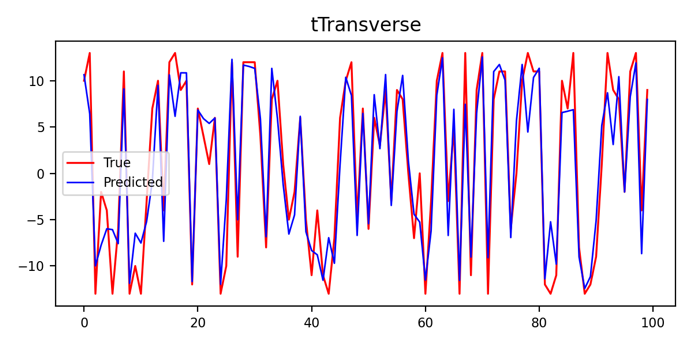 |  |

**Figure S6:** Prediction on the test set using Deep Neural Network with no PCA (256–128–64).

| 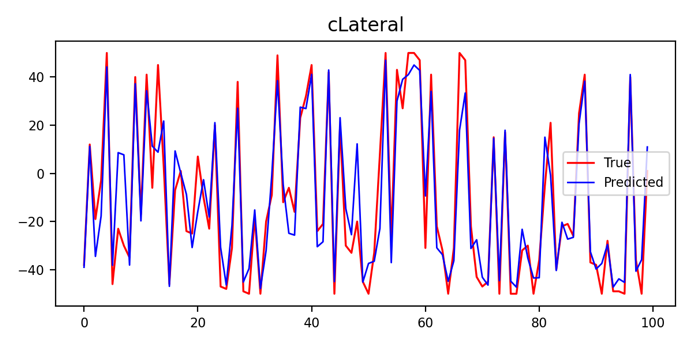 | 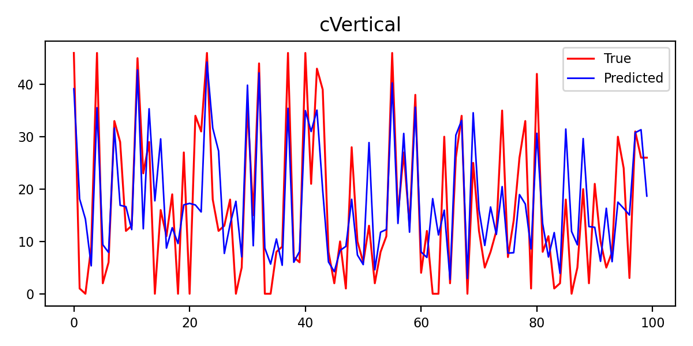 |
| --- | --- |
| 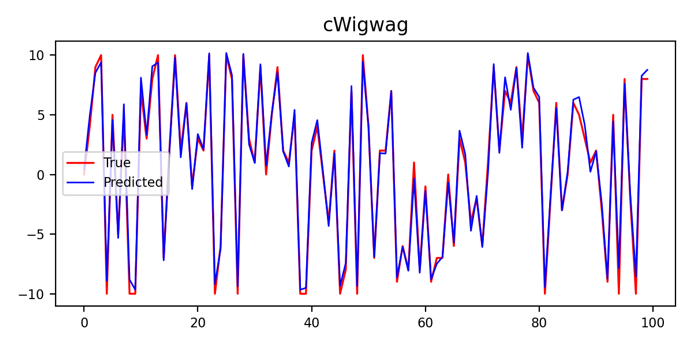 | 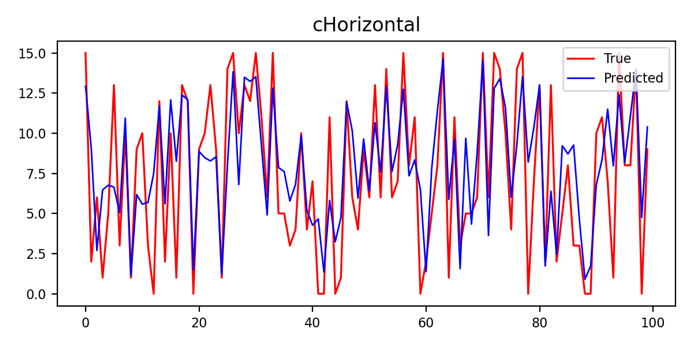 |
| 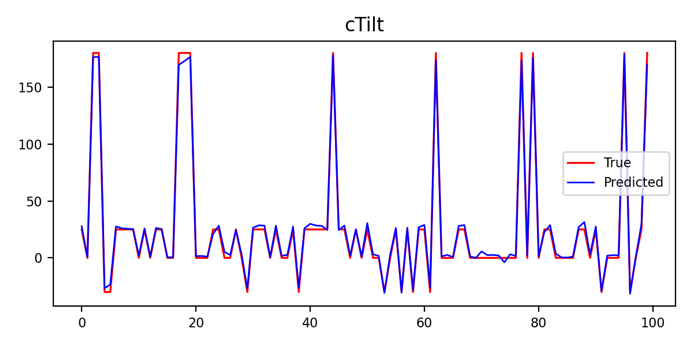 | 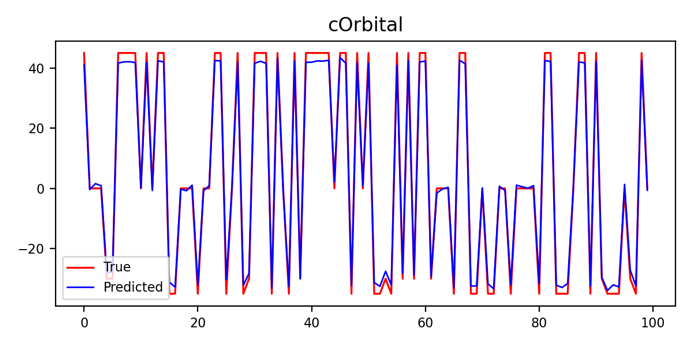 |
| 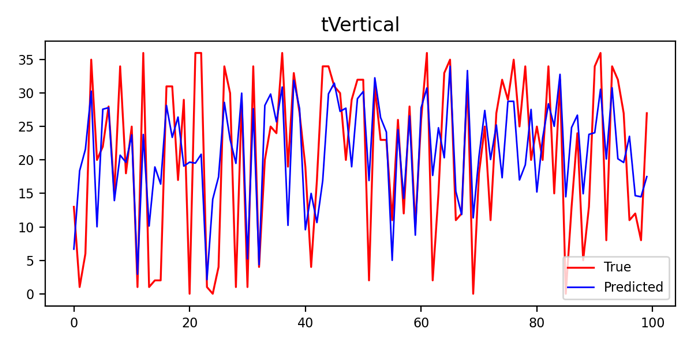 | 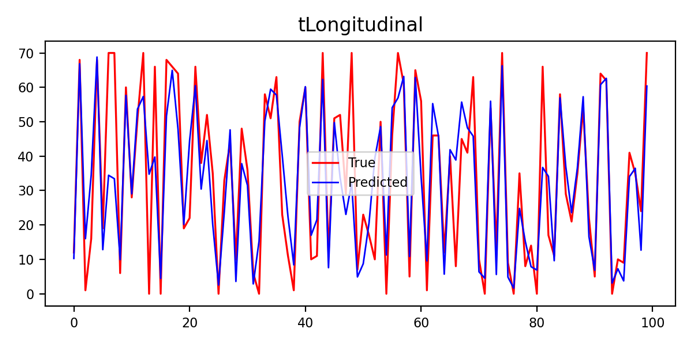 |
| 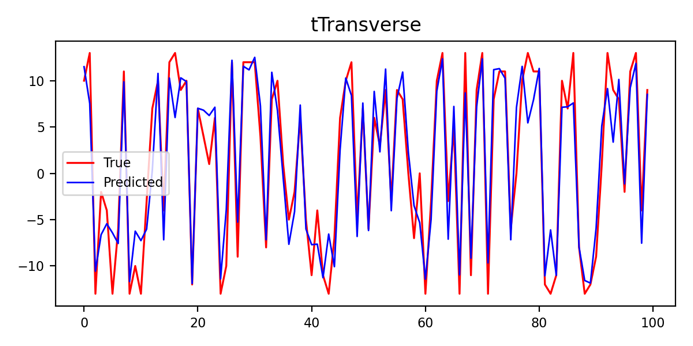 |  |

**Figure S7:** Prediction on the test set using Deep Neural Network (256–128–64) with PCA.
